# Microbe-Immune Crosstalk: Evidence That T Cells Influence the Development of the Brain Metabolome

**DOI:** 10.3390/ijms23063259

**Published:** 2022-03-17

**Authors:** Giorgia Caspani, Miranda Green, Jonathan R. Swann, Jane A. Foster

**Affiliations:** 1 Department of Metabolism, Digestion and Reproduction, Imperial College London, London SW7 2AZ, UK; g.caspani16@imperial.ac.uk (G.C.); j.swann@soton.ac.uk (J.R.S.); 2 Department of Psychiatry & Behavioral Neurosciences, McMaster University, Hamilton, ON L8S 4L8, Canada; greenm11@mcmaster.ca; 3 School of Human Development and Health, Faculty of Medicine, University of Southampton, Southampton SO16 6YD, UK; 4 St. Joseph’s Healthcare, Hamilton, ON L8N 4A6, Canada; 5 Centre for Depression and Suicide Studies, St. Michael’s Hospital, Toronto, ON M5B 1A6, Canada

**Keywords:** T cells, metabolome, microbiota, brain, *Muribaculaceae*, 5-aminovalerate, glucose-6-phosphate, butyrate, valerate, immunocompromised

## Abstract

Cross-talk between the immune system and the brain is essential to neuronal development, neuronal excitability, neuroplasticity, and neurotransmission. Gut microbiota are essential to immune system development and immune function; hence, it is essential to consider more broadly the microbiota-immune-brain axis in neurodevelopment. The gut, brain, and microbial metabolomes obtained from C57Bl/6 and T-cell-deficient mice across four developmental timepoints (postnatal day 17, 24, 28, and 84) were studied by ^1^H NMR spectroscopy. 16S rRNA gene sequencing was performed on cecal and fecal samples. In the absence of T-cells, the developmental trajectory of the gut microbiota and of the host’s metabolic profile was altered. The novel insights from this work include (1) the requirement of functional T-cells for the normal trajectory of microbiotal development and the metabolic maturation of the supra-organism, (2) the potential role for *Muribaculaceae* taxa in modulating the cecal availability of metabolites previously implicated with a role in the gut-brain axis in T-cell deficient mice, and (3) the impact of T-cell-deficiency on central levels of neuroactive metabolites.

## 1. Introduction

A paradigm shift in neuroscience and psychiatry has occurred in the last 10 years with the discovery that the trillions of microbes in our gastrointestinal tract influence brain function and behaviour. Importantly, a potential role for the microbiome and microbe-immune signalling pathways in neurodevelopment has emerged [1]. T-lymphocytes are a key mediator of this crosstalk [2,3]. While the impact of the gut microbiome on the development of the immune system is well-established, a clear understanding of how microbiota-immune signalling impacts neurodevelopment is still in its infancy.

In order to understand microbiota-immune-brain signalling, it is essential to consider the microbiota-immune interface in the gut. At the mucosal interface, exposure to and recognition of microbial antigens contribute to the establishment of a symbiotic relationship between the gut microbiota and their hosts by setting the balance between different populations of T-lymphocytes, including helper T (Th) cells and regulatory T (Treg) cells [4]. In fact, germ-free (GF) animals, born in the absence of a gut microbiota, exhibit an underdeveloped adaptive immune system, characterized by immature T and B cells [5,6]. Recently evidence is accumulating to indicate that T-lymphocytes are involved in the regulation of the gut microbiota. Severe combined immunodeficient (SCID) and non-obese diabetic SCID (NOD/SCID) mice, lacking mature B- and T-lymphocytes, exhibit abnormalities in gut microbial composition (especially an increase in *Lactobacillus*) and lower alpha diversity [7]. The same finding was also reported in T-cell deficient CD3-epsilon–/– mice, where microbiome diversity was rescued by transfer of Foxp3^+^ T-cells [8]. In mice depleted of mucosal Treg cells, the composition of the gut microbiota was significantly altered, with a decrease in mucosal-associated bacteria, *Mucispirillum schaedleri*, *Lactobacillus johnsonii*, and *Helicobacter hepaticus*, but an increase in *Alistipes* sp. and *Bacteroides uniformis* [9]. Gut microbial metabolism was also shown to be affected by T-cell deficiency, with alterations in amino acid synthesis [9] and lipid metabolism [10] in the intestine of T-cell deficient mice.

Previous work from our group based on studies of different models of combined immunodeficiencies [11,12] showed that mice depleted of T-cells via knock-out of the β and δ chains of the T-cell receptor (*TCR β–/–δ–/–)* exhibited decreased anxiety-like behaviour, but elevated baseline plasma corticosterone and enhanced gene expression changes in response to stress than C57Bl/6 (B6) mice [13,14], similar to what was previously reported in GF mice [15,16,17]. These behavioral and physiological alterations were paralleled by changes in the volume of several brain regions [13]. Recently, γδ T-cells have been shown to modulate anxiety-like behavior by releasing IL-17a at the meninges and eliciting transcriptional changes in neurons [2]. Together, these studies support a link between T-cell-microbe crosstalk and the behavioral phenotype.

In this study, a longitudinal design was used to map the temporal gut microbial and gut/brain metabolomic trajectory during postnatal development spanning from early-life to adulthood in wild type B6 and T cell deficient mice (*TCR β–/–δ–/–*) [13,14,18]. Our data showed that compared to B6 mice, *TCR β–/–δ–/–* mice had an altered trajectory of microbiome maturation, reduced alpha diversity, several differences in microbial composition, including increased abundance of *Akkermansia* and reduced abundance of *Rosburia,* and changes in gut and brain metabolite profiles. Using integrated analytical approaches, our data showed that reduced cecal and fecal butyrate levels were associated with *Muribaculaceae* taxa in *TCR β–/–δ–/–* mice. Moreover, T-cell related changes in gut microbiota and metabolome were paralled by changes in neuroactive metabolites in the brain.

## 2. Results

### 2.1. T-Cell Deficiency Altered the Developmental Trajectory of Alpha Diversity

**Figure 1:**
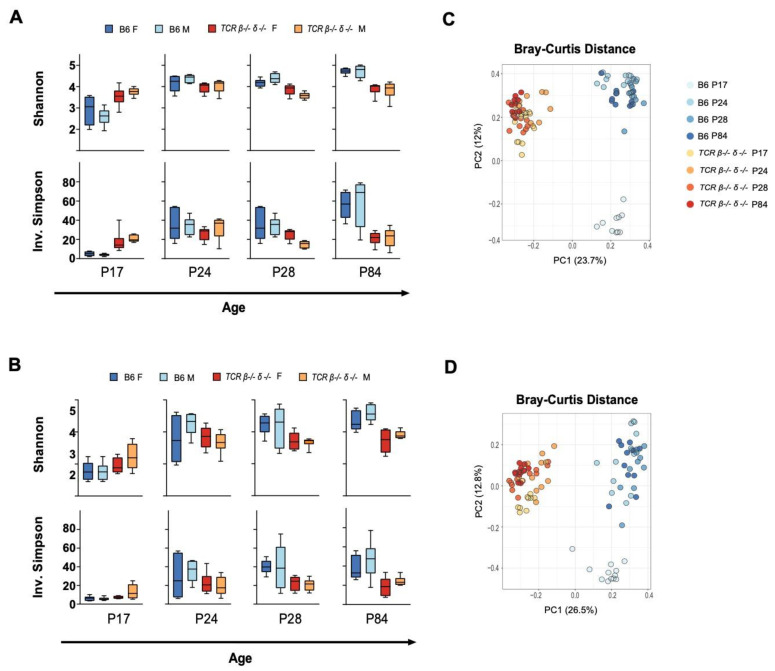
Developmental changes in alpha and beta diversity of cecal and fecal samples in T cell receptor double knock-out (*TCR β–/–δ–/–)* and C57Bl6 (B6) mice. Shannon and Inverse Simpson indices were used to calculate alpha diversity for each genotype, age and sex (*n* = 6 per genotype/age/sex). Min-max boxplots comparing alpha diversity metrics across timepoints for cecal and fecal samples are shown in (**A**,**B**), respectively. For both cecal and fecal samples, alpha diversity at P17 was higher in *TCR β–/–δ–/–* than in B6 mice (Shannon fecal *p* = 0.029, cecal *p* = 0.0005, Simpson fecal *p* = 0.029, cecal *p* < 0.0001). This trend reversed at following timepoints (P24, pre-puberty to 12 weeks, adulthood), with *TCR β–/–δ–/–* mice exhibiting lower alpha diversity than B6 mice (P24 Shannon fecal *p* = 0.133, cecal *p* = 0.038, Simpson fecal *p* = 0.08, cecal *p* = 0.39; P28 Shannon fecal *p* = 0.007, cecal *p* = 0.0002, Simpson fecal *p* = 0.007, cecal *p* = 0.0019; P84 Shannon fecal *p* = 0.0001, cecal *p* < 0.0001, Simpson fecal *p* = 0.0011, cecal *p* = 0.0002). Sex differences were not observed in alpha diversity. Principal component analysis (PCA) decomposition of Bray-Curtis dissimilarity between samples is shown in (**C**) (cecal) and (**D**) (fecal). Samples collected from B6 mice at P17 show strong clustering and separation from later timepoints, a temporal pattern not observed in the microbiota of *TCR β–/–δ–/–* mice. A significant main effect of genotype was found for both fecal and cecal samples and for all timepoints (*p* < 0.001).

Compositional differences in gut microbiota between B6 and *TCR β–/–δ–/–* mice were investigated by 16S rRNA gene sequencing. Sequencing data resulted in 2207 different amplicon sequence variants (ASV) belonging to 1095 unique assigned taxonomies at the genus level. All samples with a read count below 5000 were omitted from further analysis, resulting in a minimum and maximum number of reads per sample of 5115 and 114,609 with a median of 57,799 reads. Fecal (*n* = 6 per sex per genotype) and cecal samples (*n* = 6 per sex per genotype) were analyzed separately for all diversity measurements. The developmental trajectory of alpha diversity differed in B6 and *TCR β–/–δ–/–* mice (Figure 1). In B6 mice, alpha diversity, measured by Shannon and Inverse Simpson indices in both fecal and cecal samples, increased over postnatal development with notable stepwise increases in alpha diversity post-weaning (P24 > P17) and post-puberty (P84 > P28). In contrast, alpha diversity in *TCR β–/–δ–/–* mice was higher at P17 (pre-weaning) compared to B6 mice in both fecal and cecal samples. However, there was no additional increase in alpha diversity over the post-weaning and post-puberty time periods, resulting in reduced alpha diversity at P28 (pre-puberty) and adult (post-puberty) in *TCR β–/–δ–/–* mice compared to B6 mice. No sex differences were observed in alpha diversity (*p* > 0.05 for all timepoints).

### 2.2. Beta Diversity Analysis Revealed Significant Clustering by Genotype

Longitudinal comparison of beta diversity between genotypes was assessed through comparison of Jaccard, Bray Curtis, and Aitchison distances at the ASV level. Figure 1 shows principal component analysis of Bray-Curtis distance metrics in fecal and cecal samples (models constructed on Jaccard and Aitchison distances are shown in Supplementary Figure S1). A PERMANOVA test revealed a significant main effect of genotype for all distance metrics, timepoints, and sample types ((*p* < 0.001), indicating compositional differences between *TCR β–/–δ–/–* and B6 mice over P28 and P84) found that genotype explained the greatest amount of variation in distance metrics (Jaccard, R^2^ = 0.265; Bray-Curtis, R^2^ = 0.228; Aitchison, R^2^ = 0.263), followed by Age (Jaccard, R^2^ = 0.091; Bray Curtis, R^2^ = 0.155; Aitchison, R^2^ = 0.094), while no sex differences were observed (*p* = 0.883, 0.971, and 0.998, respectively). A similar pattern was observed in fecal samples (see all R^2^ values in Supplementary Table S1). A permutation test for homogeneity of dispersions (PERMDISP2) revealed different results across indices. A dispersion test of Jaccard distance showed no significant effect of genotype for all timepoints, in both fecal and cecal samples. However, a significant effect of genotype at P17 and P28 (*p* < 0.01) was seen in dispersion tests using Bray-Curtis distances. This was also the case for Aitchison distances, which showed significant differences in dispersion at timepoints beyond P17 for fecal and cecal samples. All dispersion test results for each distance metric are summarized in Supplementary Table S2. Although this may indicate heterogeneity of dispersion contributes to observed group differences at these timepoints, taken together with highly consistent PERMANOVA results we can generally attribute distinction of genotypes to overall differences in microbiome structure and compositional characteristics. This was reinforced visually by PCoA plots of beta diversity metrics that show strong clustering in ordination space by genotype, with significant overlap between age groups within each strain (Figure 1, Supplementary Figure S1). Interestingly, Bray-Curtis plots show a separation of P17 mice from later timepoints in B6 mice (Figure 1).

### 2.3. T-Cell Deficiency Affects the Abundance of Specific Bacterial Taxa

**Figure 2:**
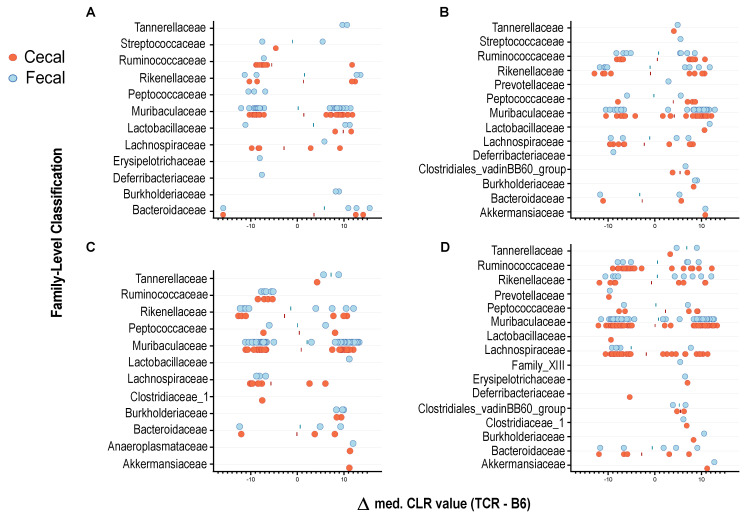
Differentially abundant taxa between C57Bl/6 (B6) and T cell receptor double knock out (*TCR β–/–δ–/–)* mice. Individual graphs represent results at (**A**) P17, (**B**) P24, (**C**) P28, and (**D**) 12 weeks. Each circle represents an individual ASV that was significantly increased or decreased between genotypes, with the median difference in CLR-transformed relative abundance plotted on the X-axis. Positive values represent taxa that are significantly more abundant in *TCR β–/–δ–/–* mice compared to B6 controls, whereas negative values represent those significantly more abundant in B6 mice.

Genotype-related differences in microbial composition were observed in cecal and fecal samples. In cecal samples, analysis revealed 45 differentially abundant taxa at P17, 74 at P24, 62 at P28, and 138 at P84. In fecal samples, analysis revealed 56 taxa significantly impacted by strain at P17, 68 at P24, 70 at P28, and 81 at P84 (Figure 2). Several ASVs were significantly reduced in abundance in *TCR β–/–δ–/–* mice compared to their B6 counterparts, as well as a number of taxa that were significantly upregulated in the context of T-cell deficiency. Interestingly, different ASVs classified as *Muribaculaceae, Lachnospiraceae*, *Ruminococcaceae*, and *Rikenellaceae* displayed opposite patterns in strain-dependent abundance, where some taxa within the same family were elevated in B6 mice while others were elevated in *TCR β–/–δ–/–* mice. This demonstrates the importance of examining differential abundance at the ASV level. Group means of relative abundance data for each genotype, sex and age are shown in Supplementary Figure S1. A small number of distinct ASVs belonging to the families *Anaeroplasmataceae, Burkholderiaceae, Erysipelotrichaceae*, and *Tannerellaceae* were uniquely elevated in *TCR β–/–δ–/–* mice. At the genus level, an increase in *Lachnoclostridium*, *Akkermansia*, *Anaeroplasma*, and *Lactobacillus*, and a decrease in *Lachnospiraceae_*NK4A136_group were observed in the cecum of *TCR β–/–δ–/–* mice. Some ASVs belonging to *Alistipes* and *Bacteroides* were increased, while others were decreased in *TCR β–/–δ–/*– mice. From P24, the butyrate-producers *Roseburia* and *Butyricicoccus* also showed a moderate reduction in abundance in *TCR β–/–δ–/–* mice relative to B6 mice.

As may be important for comparison of the current results and published or future work, our analysis allowed for direct comparison of fecal and cecal samples and assessed the impact of sample type on compositional differences. This is visualized in Figure 2. Although most differentially abundant ASVs detected in cecal samples have an obvious fecal counterpart, a small number of taxa are uniquely present in only one sample type. Overall, 44 more taxa were significantly different between genotypes in cecal samples, and only a very small number of ASVs were uniquely returned in analysis of fecal data. Although this difference may depend largely on the timepoints studied and the analysis in question, it does suggest cecal sampling provides a more robust “snapshot” of an in vivo microbiome profile and a higher sensitivity to certain taxa that may not be detectable in feces.

### 2.4. T-Cell-Deficient Mice Exhibit Altered Development of Colon, Cecal and Fecal Metabolomes

Proton nuclear magnetic resonance (^1^H NMR) spectroscopy was used to measure the metabolic profiles of intestinal (colon and cecal) and fecal samples from B6 and *TCR β–/–δ–/–* mice to identify genotype-associated differences in the gut metabolome. Based on PCA models constructed on the ^1^H NMR spectroscopy data, three cecal samples were identified as outliers (found to lie outside of the Hotelling’s T-squared 95% confidence ellipse) and removed from the data analysis. Cecal samples were not available from P17 for the metabolomic analysis.

The PCA models for each genotype identified age as the main source of variation in the colon and fecal profiles (Supplementary Figure S2). Consistent with the bacterial data, the colon and fecal metabolic profiles of B6 mice at P17 were distinct from those of later ages (P24, P28, and P84). In contrast, in the *TCR β–/–δ–/–* mice the colonic and fecal metabolic profiles of younger mice (P17) were comparable to those of older mice (P24, 28, and 84), indicating an altered developmental trajectory of both gut microbial composition and related metabolites in the absence of T-cells. Less variation in metabolic profiles was also observed in *TCR β–/–δ–/–* mice compared to B6 (Supplementary Figure S2).

**Figure 3:**
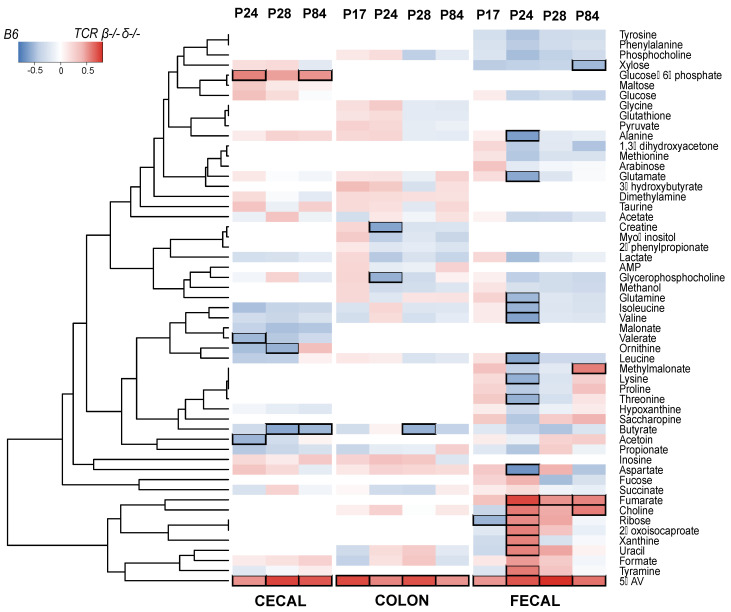
Metabolic variation of cecal, colon and fecal samples associated with genotype. The heatmap illustrates the correlation coefficient ® obtained from OPLS models at individual timepoints for cecal, colon and fecal samples. Metabolites overrepresented in (C57Bl/6) B6 mice are shown in blue, those overrepresented in T cell receptor double knock out (TCR β–/–δ–/–) mice are shown in red. Black squares indicate correlations with r > │0.5│.

Pairwise OPLS-DA models were constructed to compare the profiles of age-matched B6 and *TCR β–/–δ–/–* mice. The cecal metabolic profile of B6 and *TCR β–/–δ–/–* mice was significantly different at P28 (Q^2^Y = 0.537, *p* = 0.001) and P84 (Q^2^Y = 0.460, *p* = 0.001), but not in early life (P24). From each discriminant analysis model, the correlation r between the metabolites and the class membership was extracted and represented in the heatmap in Figure 3. The gut microbial metabolite 5-aminovalerate was markedly more abundant in the cecum of *TCR β–/–δ–/–* mice compared to the B6 group at P28 (r = 0.738) and P84 (r = 0.687). In contrast, the microbial-derived short-chain fatty acid (SCFA) butyrate was lower in *TCR β–/–δ–/–* mice compared to the B6 mice at P28 (r = −0.581) and 84 days (r = −0.511). Butyrate is important to intestinal barrier integrity. In addition, both 5-aminovalerate and butyrate can be produced by microbial metabolism of essential amino acid lysine [18,19,20]. The lack of T-cells also resulted in lower amounts of cecal ornithine at P28 (r = −0.537) and higher amounts of glucose-6-phosphate at 84 days (r = 0.513), metabolites related to amino acid metabolism and glycolysis. In the feces, metabolic variation was identified between the *TCR β–/–δ–/–* and B6 mice at P24 (Q^2^Y = 0.373, *p* = 0.037) and P28 (Q^2^Y = 0.425, *p* = 0.032) with 5-aminovalerate and fumarate present in greater abundance in *TCR β–/–δ–/–* mice. In addition, uracil (r = 0.562), xanthine (r = 0.560), 2-oxoisocaproate (r = 0.568), choline (r = 0.590), ribose (r = 0.540), and tyramine (r = 0.587) were more abundant in *TCR β–/–δ–/–* mice at P24. The branched-chain amino acids valine (r = −0.600), leucine (r = −0.586), and isoleucine (r = −0.535), the amino acids alanine (r = −0.621), lysine (r = −0.545), threonine (r = −0.542), and glutamine (r = −0.508), and the excitatory neurotransmitters glutamate (r = −0.578) and aspartate (r = −0.665) were lower at P24 in *TCR β–/–δ–/–* mice relative to B6.

Clear differences were observed in the colonic metabolic profiles of *TCR β–/–δ–/–* and B6 mice at P17 (Q^2^Y = 0.465, *p* = 0.001), P24 (Q^2^Y = 0.392, *p* = 0.003), P28 (Q^2^Y = 0.257, *p* = 0.020), and P84 (Q^2^Y = 0.462, *p* = 0.001). Compared to the B6 animals, the colonic contents of *TCR β–/–δ–/–* mice contained greater amounts of 5-aminovalerate at all developmental timepoints, being particularly pronounced at P17 (r = 0.726). At P24, the abundance of glycerophosphocholine (r = −0.542) and creatine (r = −0.592) were lower in the *TCR β–/–δ–/–* mice compared to the B6 mice and at P28 butyrate was also lower (r = −0.537). Supplementary Figure S3 shows the coefficient plots of the correlation between metabolites and class membership (i.e., genotype) for each timepoint and each sample type.

### 2.5. Abundance of Muribaculaceae Is Linked to Cecal Concentration of Microbial Metabolites

**Figure 4:**
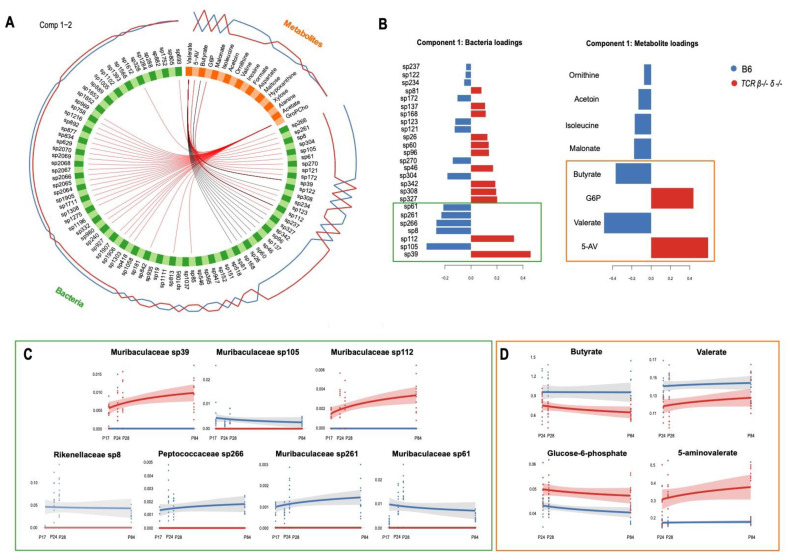
Integration of metabolic and bacterial abundance data. (**A**) Circos plot of the multi-block relationships across two principal components associated with T-cell deficiency. The correlation between variables of different blocks (*metabolites* in orange, *bacteria* in green) are shown with red (if positive association) or black (if negative association) inner lines. The expression level of each variable is also shown as the blue (expression level in B6) and red (expression level in T cell receptor double knock out (*TCR β–/–δ–/–)* mice) lines on the outside of the circle. Only absolute correlations higher than 0.6 are shown. (**B**) Loading weights showing the best discriminating features on PC1 for both *bacteria* (left) and *metabolites* (right) blocks. The X-axis represents the coefficient weight of the variables listed on the Y-axis. In blue are the variables that have the maximum level of expression in B6, in red those maximally expressed in *TCR β–/–δ–/–*. (**C**) Trajectories of the top seven bacterial taxa (as per loading weights in panel B) across developmental timepoints in *TCR β–/–δ–/–* (red) and B6 mice (blue), supporting the correlations highlighted by the Circos plot. (**D**) Trajectories of the top four metabolites (as per loading weights in panel B across developmental timepoints in *TCR β–/–δ–/–* (red) and B6 mice (blue), supporting the correlations highlighted by the Circos plot. Shaded areas represent the confidence interval around smooth lines obtained by constructing a linear model with the formula y ~ log(x), where x = age and y = metabolite/bacterial abundance.

To examine the association between gut microbial features and their metabolites, and test for their ability to discriminate between B6 and *TCR β–/–δ–/–* mice, relative abundance and all metabolic data (not limited to discriminatory features) obtained from cecal samples were integrated using a supervised classification algorithm called Data Integration Analysis for Biomarker discovery using Latent cOmponents (DIABLO, mixOmics package). An exploratory partial least squares regression (PLS) approach was used to determine the global correlation (i.e., correlation between first principal components) between metabolic and bacterial phenotypes. The resulting correlation coefficient (r = 0.83) was used to tune the design matrix, which specifies the connection strength between data blocks. The tune.block.splsda() function was used for feature selection and the final multi-block model was constructed on 18/27 metabolites and 83/1532 ASV (listed in Supplementary Table S3). A permutation test based on cross-validation with 999 iterations demonstrated the validity of the model (*p* = 0.001). The model confirmed clear differences in the bacterial and metabolic signatures of *TCR β–/–δ–/–* and B6 mice. The ASVs *Muribaculaceae* sp39 and sp112, *Peptococcaceae* sp327 and sp342, *Ruminococcaceae* sp308 (expanded in *TCR β–/–δ–/–*) and *Muribaculaceae* sp105, sp261 and sp61, and *Rikenellaceae* sp8 and *Peptococcaceae* sp266 (expanded in B6) had the highest loading weights along principal component (PC) 1, suggesting that these bacterial taxa account for the largest source of variance between genotypes. Similarly, metabolites 5-aminovalerate and glucose-6-phosphate (upregulated in *TCR β–/–δ–/–*) and valerate, butyrate, malonate, isoleucine, acetoin, and ornithine (upregulated in B6) drove the separation of the metabolic profiles of *TCR β–/–δ–/–* mice from that of their B6 counterparts (Figure 4). Filtering the correlations with a threshold of r ≥│0.6│, the Circos plot highlighted a number of associations between individual bacterial taxa and metabolites (Figure 4). Along PC1, mice lacking T cells exhibited higher abundance of *Muribaculaceae* sp39 and sp112, both positively correlated to 5-aminovalerate and glucose-6-phosphate (also higher in *TCR β–/–δ–/–*), but negatively correlated to valerate (higher in B6). A negative correlation between *Muribaculaceae* sp39 and butyrate (higher in B6) was also highlighted. While 5-aminovalerate, glucose-6-phosphate, and butyrate were only related to the concentrations of *Muribaculaceae* sp112 and sp39, the SCFA valerate was found to be strongly correlated to several other taxa (including *Rikenellaceae, Ruminococcaceae,* and *Peptococcaceae* families). The original similarity matrix used to build the Circos plot can be found in Supplementary Table S4. Along PC2, the metabolite glycerophosphocholine was positively linked to 30 bacterial taxa (belonging to the *Muribaculaceae, Lachnospiraceae, Rikenellaceae, Ruminococcaceae*, and *Peptococcaceae* families), all of which were more abundant in B6 relative to *TCR β–/–δ–/–* mice. However, glycerophosphocholine exhibited poor discriminatory ability between the genotypes and was not investigated further. The Circos plots built on individual components are shown in Supplementary Figure S4. Consistent with what was highlighted by the multi-omic model, the ASVs corresponding to *Muribaculaceae* sp39 and sp112 and the positively correlated gut-microbial metabolite 5-aminovalerate were absent in B6 mice, while abundant in *TCR β–/–δ–/–* mice across development (Figure 4). A remarkably similar trajectory was observed between *Muribaculaceae* sp39 and sp112 and 5-aminovalerate, all increasing with age in *TCR β–/–δ–/–* mice. In contrast, *Muribaculaceae* sp105, 261 and sp61, *Rikenellaceae* sp8 and *Peptococcaceae* sp266 were absent in *TCR β–/–δ–/–* mice but abundant in B6 mice at all ages. The metabolites butyrate and valerate were lower, while glucose-6-phosphate was elevated in *TCR β–/–δ–/–* mice compared to B6 throughout development.

### 2.6. T-Cell-Deficient Mice Exhibited Altered Development of Cortical and Hippocampal, but Not Hypothalamic, Metabolomes

PCA plots were constructed separately on the brain metabolic profiles of B6 and *TCR β–/–δ–/–* mice to observe the metabolic variation associated with age. Five cortical samples were identified as outliers, possibly due to low tissue volume, and were excluded. In both genotypes, the metabolic variation along PC2 was driven by age (Supplementary Figure S5), demonstrating a moderate effect of developmental timepoint on the brain metabolome. Consistent with the microbial data and colon/fecal metabolic data (Supplementary Figure S2), the metabolic signatures of the cortical and hippocampal regions of B6 mice at P17 were distinct from those of older mice (P24-P84). This was not seen with the *TCR β–/–δ–/–* mice, which did not exhibit the same pre- and post-weaning clustering. Interestingly, this pattern was not observed in hypothalamic samples, where for both B6 and *TCR β–/–δ–/–* mice, the metabolic signature of P17 mice overlapped with that of older mice (Supplementary Figure S5C).

### 2.7. T-Cell Deficiency Affects the Expression of Neuroactive Metabolites

In the brain, the abundance of five metabolites showed clear differences across developmental timepoints for B6 and *TCR β–/–δ–/–* mice (Supplementary Figure S6). A two-way ANOVA confirmed that genotype had a significant main effect on aspartate and glycerophosphocholine in all brain samples. A significant effect of genotype was also found for acetate, GABA, and phosphocholine in both hippocampus and hypothalamus. A significant combined effect of genotype and age was found for cortical acetate and glycine (Table 1). Interestingly, the four cecal metabolites that emerged from the multi-omics integration analysis (5-aminopentanoate, glucose-6-phosphate, butyrate, and valerate) were found to be significantly correlated with several cortical, hippocampal, and hypothalamic metabolites (Pearson correlation). These results are shown in Supplementary Figure S6.

## 3. Discussion

The present study demonstrated that T-cell deficiency alters the development of the gut microbiota and of the host’s gastrointestinal and brain metabolome. While the influence of the microbiome on immune development is well-established [4], the results of the current study demonstrate clearly the bidirectional nature of this relationship and identify several key taxa that are important to microbiota-immune crosstalk during postnatal development. Notably, the gut-related changes in microbial composition, diversity, and metabolite profile were accompanied by parallel changes in the hippocampal and hypothalamic metabolome. From a developmental perspective, it is interesting to note that most significant T-cell related changes in gut, microbial, and brain metabolites developed in the fourth week of postnatal development, post-weaning.

While a role for microbiota in immune development, and in particular T-cells, has been previously established, the results of the current study suggest that T-cell to microbe signalling in the postnatal period is required for normal microbiota maturation, diversity, and composition. In particular, *TCR β–/–δ–/–* mice showed higher microbial diversity initially at P17 but lower diversity at all later developmental stages. A reduction in alpha-diversity has been previously reported in mouse models of combined or T-cell-specific immunodeficiency [7,8,9], and was recently confirmed in *TCR β–/–δ–/–* mice in an independent study (Francella et al., in prep). In wild type mice, T-cell progenitors undergo exponential proliferation in the thymus, which reaches maximal size at P18 [21]. These findings indicate that, in the absence of host-orchestrated immune signalling during this critical developmental window, the colonization trajectory of the maturing microbiota was modified. The increased diversity exhibited in at P17 in *TCR β–/–δ–/–* mice may reflect accelerated microbiota maturation. In addition, B6 mice exhibited a compositional and metabolic shift between P17 and P24 that is likely to represent the expansion, maturation, and stabilization of the microbiome at weaning. Interestingly, in the absence of T-cells, P17 mice had similar compositional and metabolic profiles than older mice, suggesting that an early signal from T-cells to microbes is necessary to support normal microbiota and metabolic development into adulthood. The presence of gut microbes is known to be essential for the maturation of functional T-cells [22]. These findings suggest that this relationship is reciprocal, and we propose that bidirectional communication between microbes and T-cells may influence the trajectory of development and maturation of both systems. Given the overlapping developmental window of T-cells with that of the gut microbiota and of the central nervous system, it was not surprising that the impact of T-cell deficiency spanned the entire gut-brain axis. Previous work has demonstrated a range of neurodevelopmental delays (e.g., righting reflex and ultrasonic vocalizations) emerging as early as P4-6 in *TCR β–/–δ–/–* mice (Francella et al., in prep). Similarly, the presence of gut microbial metabolites in the mouse brain has been shown as early as P3 [23]. Thus, the present work expanded on the limited evidence for a role of T-cells in shaping gut microbiome maturation and their influence on neurodevelopment.

The reorganization of the microbial community in T-cell deficient mice was accompanied by downstream effects on host metabolic signature. The Bacteroidales family *Muribaculaceae*, previously *S24-7* [24] and *Candidatus homeothermaceae* [25], known to be involved in the degradation of complex carbohydrates (*i.e.,* α-glucan, host or plant glycans) and propionate production [26,27,28], showed the strongest response to T-cell deficiency. Some ASVs within this family were more abundant in *TCR β–/–δ–/–* mice, while others were less abundant compared to the B6 mice. Our integrated analysis suggested that these compositional changes may drive the observed functional alterations, as the increase in 5-aminovalerate and glucose-6-phosphate in parallel with reduced butyrate and valerate was associated with an increase in two *Muribaculaceae* ASVs, sp39, and sp112. These ASVs may correspond to what Smith et al. (2020) referred to as “acarbose responders”, with members of the *Muribaculaceae* family able to produce glucose-6-phosphate from starch fermentation. Thus, higher relative abundance of these *Muribaculaceae* species may lead to the increase in cecal glucose-6-phosphate in the *TCR β–/–δ–/–* mice. The present work also demonstrated a reduction in butyrate-producing bacteria in *TCR β–/–δ–/–* mice, including *Roseburia*, *Butyricicoccus* and *Lachnoclostridium*. NMR spectroscopic analysis of cecal samples confirmed lower amounts of the SCFAs butyrate and valerate, as well as higher amounts of their precursors, glucose-6-phosphate and 5-aminovalerate, in *TCR β–/–δ–/–* mice. The GABA homologue 5-aminovalerate (also known as 5-aminopentanoate [18,19]), butyrate [20], and valerate [29] can be produced by microorganisms in the gut from the essential amino acid lysine, which can be both dietary and microbial in origin. Thus, in the absence of T-cells, a rearrangement in microbial composition modulated the biochemical activity of the microbiota and the flow of these microbial signals to the host, which we suggest may lead to the preferred utilization of lysine for 5-aminovalerate over SCFA production. Some *Muribaculaceae* species (e.g., *Muribaculum intestinale*, 99.76% sequence homology with sp123 [30]) are known to express the enzyme saccharopine dehydrogenase, which diverts lysine towards saccharopine as a degradation byproduct [31]. Thus, the reduction in *Muribaculaceae* sp123 may explain the higher abundance of 5-aminovalerate in the cecum of *TCR β–/–δ–/–* mice. In addition, certain *Clostridia* species can metabolize 5-aminovalerate to valerate and other SCFAs [32]. A reduction in *Clostridia* species has been reported in *TCR β–/–δ–/–* mice in the current work (e.g., *Lachnospiraceae_NK4A136_group*), as well as in a separate study (Francella et al., in prep) and in mice lacking both T- and B-lymphocytes [7].

SCFA have known immunomodulatory properties, both in the gastrointestinal tract and in the central nervous system. In particular, butyrate exerts protective effects by modulating barrier integrity [33,34] and mucus production [35], and contributes to the maintenance of an anaerobic environment in the large intestine [36]. More importantly, it has been shown to promote the differentiation of CD4+ naïve T-cells into colonic regulatory T-cells (Tregs) via epigenetic mechanisms involving the inhibition of histone deacetylases [37,38,39]. The results of this study suggest that T-cells can signal back to the microbiome to modulate the metabolic environment at the intestinal mucosa. Supporting evidence for its bidirectional nature comes from findings that T-cell recruitment can induce transcriptional changes in both the microbiota, limiting SCFA production [40], and in the intestinal epithelium, favoring mucus expression [41] and modulating AMP release and lipid metabolism [10].

In addition to a detailed map of the impact of T-cell deficiency on the microbial-metabolomic profile in the gut, this study demonstrated T-cell deficiency on development of the brain metabolome, with higher abundance of GABA, glycine, aspartate, acetate and glycerophosphocholine in the brains of *TCR β–/–δ–/–* mice across the lifespan. A role for T-cells in modulating brain development has been established, with previous studies showing changes in the volume of several brain regions in *TCR β–/–δ–/–* mice [13], paralleled by decreased anxiety-like behaviour, but elevated basal cortisol in *TCR β–/–δ–/–* mice [13,14]. Further, IL-17a released by meningeal γδ T-cells have also been shown to modulate anxiety-like behavior [2], and effect that may be mediated by microglia by inducing phagocytosis of neural progenitor cells [42]. The observation that both T-cells [43] and gut bacteria [44] are necessary to support microglia maturation suggests that the next key step is to determine mechanistically how these central immune cells mediate the brain abnormalities observed in the absence of a functional T-cell-microbe crosstalk. It is also of interest to consider how the interplay between T-cells and microglia in synaptic remodeling [43,45] may explain brain volume abnormalities observed in *TCR β–/–δ–/–* mice.

While the current study did not directly examine IL17, evidence for a dysfunctional T-cell-microbe crosstalk in CNS disorders was provided in the context of experimental autoimmune encephalomyelitis (EAE, a model of multiple sclerosis, [46]) and maternal immune activation (MIA, a model of autism, [47,48,49]), where induction of the T helper 17 (Th17)/IL-17a pathway by members of the gut microbiota elicited cortical and behavioral abnormalities. In the absence of neuronal IL-17a signalling, an upregulation of genes involved in GABA neurotransmission was also observed [2]. While not directly tested here, this is consistent with the changes in GABA in the present study in the frontal cortex, hippocampus and hypothalamus. Additional work is needed to demonstrate whether such alterations are the molecular basis for the abnormalities in anxiety-like behavior in *TCR β–/–δ–/–* mice [13,14]. In addition, the reported negative association between anxiety and fecal abundance of *Muribaculaceae* in humans [50] is intriguing and we suggest that this behavioral trait may be responsive to the changes in the microbiota of *TCR β–/–δ–/–* mice.

In conclusion, this paper shows that bidirectional T-cell-microbe communication is a component of normal microbiota-immune development, and highlights specific bacteria and metabolites that are key to this crosstalk. In addition, it demonstrates that T-cell deficiency impacts the concentrations of brain metabolites, possibly via a rearrangement in the microbial community and downstream changes in the host’s metabolic signature. Further work will need to determine the signalling mechanism by which such changes may lead to abnormalities in brain anatomy and behavior reported in *TCR β–/–δ–/–* mice. A key future verification of the impact of T cells on the development of the microbiota-immune brain axis would be to demonstrate that T cell replacement during early life would rescue the microbial or metabolic phenotype. The key findings here have important translational value as researchers move to consider how microbiota-immune relationships influence the trajectory of development of the microbiome and impact human brain development [51]. Moreover, a more mechanistic understanding of the molecular entities on the microbe side and on the host side of this crosstalk in animal models and in people has the potential to provide novel biomarkers and new targets for microbiota-based therapies that can foster healthy microbiome maturation, in parallel with healthy brain development.

## 4. Materials and Methods

### 4.1. Animals

T-cell receptor double knock-out mice (*TCR β–/–δ–/–*) on a C57Bl/6 background and B6 controls (*n* = 6 per sex per genotype per time point) were used in this study. Lack of functional T-cells was due to genetic knockout of both β and δ chains of the T-cell receptor (*TCR β–/–δ–/–*) [52]. The mice were provided by Dr Andrew McPherson at McMaster University, while C57Bl/6 (B6) mice were initially purchased from Charles River (Kingston, USA) and bred in house at St. Joseph’s Healthcare animal facility. The mice were maintained in specific-pathogen-free housing in sanitized cages with filter bonnets, under a 12 h light–12 h dark cycle, with lights on at 5 AM. Food and water were available *ad libitum*. At weaning (post-natal day (P) 21), pups were caged by sex with 2–4 littermates per cage, for a total of 18 litters. At P17, P24, P28, and P84, brains were removed and brain regions collected by gross dissection including hypothalamus, hippocampus, and cortex. Brain tissues, colon, cecal, and fecal samples were immediately frozen by placing pre-weighed tubes with tissue added on dry ice and then stored at −80 °C until processing. One fecal and cecal sample per sex per genotype per timepoint was processed at McMaster University for 16S rRNA sequencing and the remaining samples were shipped to Imperial College London (UK) and stored at −80 °C until analysis. All mice were drug- and test-naïve and weighed 14.0 g on average. All experimental procedures were approved by the Animal Research Ethics Board of McMaster University, in accordance with the guidelines of the Canadian Council on Animal Research.

### 4.2. 16S rRNA Sequencing

Bacterial DNA was extracted from cecal and fecal samples using methods previously described with some modifications [53]. In brief, samples were first transferred to screw cap tubes containing 2.8 mm ceramic beads, 0.1 mm glass beads, GES and sodium phosphate buffer as described. Samples were then bead beat and centrifuged, and the supernatant was further processed using the MagMAX Express 96-Deep Well Magnetic Particle Processor (Applied Biosystems) with the Multi-Sample kit (Life Technologies #4413022). 16S rRNA gene sequences were amplified according to published protocols with modifications outlined by Whelan and colleagues [54,55], using PCR primers specific for the variable 3 (v3) and variable 4 (v4) regions of the 16S ribosomal RNA (rRNA) encoding gene (341f-CCTACGGGNGGCWGCAG and 802r-GGACTACNVGGGTWTCTAAT′). For this process, 50 ng of DNA was used as template with 1U of Taq polymerase (Thermofisher, Waltham, MA, USA), 1× buffer, 1.5 mM MgCl2, 0.4 mg/mL BSA, 0.2 mM dNTPs, and 5 pmol each of 341F and 806R Illumina adapted primers. The reaction was carried out with an initial step at 94 °C for 5 min, followed by 5 cycles of 94 °C for 30 s, 47 °C for 30 s and 72 °C for 40 s. Another 25 cycles were executed at 94 °C for 30 s, 50 °C for 30 s, and 72 °C for 40 s, with a final extension of 72 °C for 10 min. Resulting PCR products were visualized on a 1.5% agarose gel to verify amplicon size. Positive amplicons were normalized using the SequalPrep normalization kit (ThermoFisher #A1051001) and sequenced on the Illumina MiSeq platform at the McMaster Genomics Facility.

### 4.3. Sample Preparation for Metabolomics

^1^H NMR spectroscopy was performed on aqueous extracts obtained from brain, gut (cecum and colon) and fecal tissue. Approximately 30 mg of tissue was homogenized in 300 μL of ice-cold CHCl_3_/MeOH (2:1 *V*/*V*) using a TissueLyser from Qiagen (West Sussex, UK), in a 2 mL homogenization tube containing 1 mm zirconia beads (Fisher Scientific, Hampton, NH, USA) for 60 s at 6500 rpm. After addition of 300 μL of H_2_O, the homogenate was vortexed, and then centrifuged at 17,000× *g* for 10 min at 4 °C (Microstar 17R, VWR International, Radnor, PA, USA). The aqueous and lipid layers of the supernatant were collected separately. To maximize metabolite recovery, the extraction was repeated by resuspending the pellet in 300 μL of ice-cold CHCl3/MeOH (2:1 *V*/*V*) and 300 μL of water. This was vortexed and centrifuged before the aqueous and lipid layers were separated and combined with the first extraction. The aqueous extracts were left in a SpeedVac overnight at 45 °C to remove MeOH from the extract (Concentrator Plus, Eppendorf, Stevenage, UK). Samples were stored at −40 °C until the day of NMR. On the day of NMR, samples were reconstituted using 60 µL of phosphate buffer (pH 7.4) and 540 µL of D_2_O:H_2_O (9:1 *V*/*V*) containing 1 mM of TSP. The samples were vortexed to ensure reconstitution and centrifuged at 9000× *g* rpm for 10 min at 4 °C. Then, 550 µL of the supernatant was transferred to 5 mm outer diameter NMR tubes. After thawing, fecal pellets were weighed at approximately 30–50 mg. Zirconia beads and 300 µL of H_2_O was added and samples were homogenized for 2 cycles of 6500 rpm over 45 s each. Samples were centrifuged at 17,000× *g* for 20 min at 4 °C. 180 µL of the supernatant was transferred to a new Eppendorf tube and 20 µL of urine buffer was added. Samples were vortexed and centrifuged again before 180 µL of the supernatant was transferred to 3 mm NMR tubes using an eVol digital analytical syringe.

### 4.4. Metabolic Phenotyping

Aqueous extracts were obtained from brain, gut wall, cecal, and fecal samples using our established methods (see Supplementary Information for detailed methods). The aqueous tissue extracts were measured on a 600 MHz Bruker Avance III spectrometer (Bruker BioSpin, Billerica, MA, USA) operating at a constant temperature of 300 K. A standard one-dimensional pulse sequence was used: RD-90°-t-90°-tm-90°-acquire free induction decay (FID) [t = 3 μs]. Irradiation of the water signal was performed during the relaxation delay (RD) of 2 s and during the mixing time of 100 ms. The field frequency was locked on D_2_O solvent, and 64 scans were recorded. After acquisition, ^1^H NMR spectra were automatically phase and baseline corrected, and calibrated to TSP at δ 0.0, using TOPSPIN version 3.5 (Bruker BioSpin, Billerica, MA, USA). The spectra were exported into MATLAB (MathWorks) using the Imperial Metabolic Profiling and Chemometrics Toolbox for Spectroscopy or IMPacTS (https://csmsoftware.github.io/docs/impacts/index.html, last accessed 31 January 2021). Signals from TSP and water resonances were removed and automatic alignment was performed using a recursive segment-wise peak alignment (RSPA) method developed at Imperial College [56]. Prior to analysis, the data was log-transformed and normalized using total area normalization, to compensate for differences in the volumes of the tissue extracts. Multivariate analysis was performed on mean-centered ^1^H NMR spectroscopic profiles. Assignment of metabolites was performed with the aid of a combination of two-dimensional homonuclear NMR spectroscopy (J-resolved spectroscopy, correlation spectroscopy, total correlation spectroscopy), statistical total correlation spectroscopy [57] and an in-house database built from authentic standards.

### 4.5. Data Analysis

#### 4.5.1. 16S rRNA Analysis

The resulting amplicons were cleaned, quantified and sequenced on the Illumina MiSeq platform, before undergoing further processing as previously reported [55]. Read trimming was performed using Cutadapt [58], followed by filtering, dereplication, sample inference, chimera identification, and the merging of paired-end reads using DADA2, version 1.16 [59]. Amplicon sequence variants (ASVs) were generated from the sequences and assigned taxonomic classification using the Ribosomal Database Project (RDP) classifier and the SILVA 2017 reference database [60]. Alpha diversity and beta diversity analyses were completed using the vegan package in R version 4.0.2 [61]. Alpha diversity metrics included the Inverse Simpson index and the Shannon index. Differences between strains were assessed using a Kruskal Wallace H test at each timepoint, with a significance cut-off of *p* < 0.05. Beta diversity between samples was explored using principal coordinate analysis (PCoA) with Jaccard, Bray-Curtis, and Aitchison distance metrics applied to ASV count data. Genotype-based differences were assessed at each postnatal day using PERMANOVA (1000 permutations) and a homogeneity of dispersion test via the betadispr() function. Differentially abundant taxa between genotypes were assessed using the ADLEx2 package, version 1.22.0 [62]. This workflow accounts for the compositional nature of microbiome data using a centered log-ratio (CLR) transform, implemented via the aldex.clr() function, which maintains taxonomic ratios within a sample while removing the interdependency of bacterial abundances that arise from relative population measurements. This transform introduces the convenient property of scale invariance, which accounts for discrepancies in read count between samples and produces the same species ratios regardless of sequencing depth [63]. The aldex.clr function was used with 16 Monte Carlo instances sampled from a Dirichlet distribution to generate a distribution of probabilities for each taxon consistent with the observed data. Following transformation and group-wise comparison, differentially abundant taxa were determined by a Benjamini-Hochberg corrected *p*-value < 0.05 for a Kruskal-Wallace test, and an effect size with an absolute value > 1 (using the aldex.kw() and aldex.effect() functions, respectively). Statistical details, including exact value of *n*, can be found in the respective results section.

#### 4.5.2. Metabolomic Analysis

The multivariate data analysis was carried out using MATLAB 2018b (MathWorks) and scripts developed in-house [64]. The NMR data underwent quality control and spectra with poor water suppression were excluded from the analysis. Principal component analysis (PCA) was used to explore the intrinsic metabolic variability in the population and identify outliers. Age-associated variation in the metabolome was investigated separately for B6 and *TCR β–/–δ–/–* mice using an orthogonal projection to latent structures-discriminant analysis (OPLS-DA) approach with the ^1^H NMR spectral data serving as the descriptor matrix and age as the response variable (Y predictor). Biochemical variation between B6 and *TCR β–/–δ–/–* mice was investigated at each sampling point by setting genotype as the response variable (Y predictor). Seven-fold cross-validation was carried out to determine the predictive ability of the OPLS models. Models with positive R^2^ and Q^2^, and with a permutation testing *p* < 0.05 (999 permutations) were considered significant. The influence of individual metabolites to the model were assessed based on their correlation with class membership (e.g., B6 vs. *TCR β–/–δ–/–*). Discriminatory metabolites with mean r > |0.5| were identified as being differentially regulated between genotypes. Statistical details, including exact value of *n*, can be found in the respective results section.

#### 4.5.3. Multi-Block Discriminant Analysis with DIABLO

Metabolomic and 16S rRNA gene sequencing data were integrated with Data Integration Analysis for Biomarker discovery using Latent cOmponents (DIABLO, mixOmics package version 6.12.2). DIABLO is an extension of canonical correlation analysis (CCA) and Projection to Latent Structures (PLS), where principal components are built across blocks to maximize the covariance among them and with the outcome Y (i.e., B6 vs. *TCR β–/–δ–/–*). The result is a signature of correlated variables across multiple datasets measured on the same individuals that discriminate an outcome of interest [65,66]. The procedure requires tuning of three parameters: the design matrix, number of components and number of variables in each dataset to include in the final model. The resulting circos plot is built on a similarity matrix (an approximation of a Pearson correlation matrix), where each entry is calculated as the correlation between two variables’ projections on the principal component. The design matrix specifies how datasets should be correlated, with values between zero (not correlated) and one (datasets are fully correlated). We tuned the design matrix by building multi-block PLS models on metabolic and bacterial datasets. The relative abundances of cecal metabolites were calculated from the integrals of non-overlapped peaks identified in the normalized NMR spectra. Abundances were log-transformed to account for a left-skewed distribution. The global correlation between data sets was obtained by calculating the correlations between the variates on principal component 1 for the dataset pair. The model was constructed using the following design C:

$$C=\left[\begin{array}{c} \;0\;0.825\;\\ \;0.825\;0 \end{array}\right]$$

The optimal number of components to include in the model was assessed with perf.diablo() run with 10-fold cross validation and 50 repeats. To identify the optimal number of variables to retain in the model, the function tune.block.splsda() was run with 10-fold cross validation and 10 repeats using centroids distance, with a sequence of variables ranging from 8 to 20 for metabolites and 25 to 800 for bacteria. This resulted in 18 variables for metabolites and 83 for bacteria. Model performance was evaluated using classification error rates and a permutation test with 999 iterations (performed with R package RVAideMemoire [67] to determine the significance of the model (alpha threshold = 0.05). Across components, the explained variance was 24.5% for metabolites and 9.4% for ASVs. Associations with an absolute correlation coefficient higher than 0.6 were shown and discussed. Statistical details, including exact value of *n*, can be found in the respective results section.

## Figures and Tables

**Table 1:** Results of 2-way ANOVA against genotype and age for brain metabolites.

	Cortex		Hippocampus		Hypothalamus	
	Genotype	Genotype/Age	Genotype	Genotype/Age	Genotype	Genotype/Age
Aspartate	0.003	-	<0.001	-	0.002	-
Glycerophosphocholine	0.001	-	0.008	-	<0.001	-
Acetate	-	0.049	0.035	-	0.030	-
GABA	-	-	0.044	-	0.023	-
Glycine	-	0.008				
Alanine	-	-	0.004	-	-	-
Phosphocholine	-	-	0.037	-	0.004	-
IMP	-	-	-	<0.001	-	-
Taurine	0.011	-	-	-	0.007	-
Glutamate	-	-	-	-	0.042	-
Myo-inositol	-	-	-	-	0.013	-
3-hydroxyisovalerate	-	-	-	-	0.039	-
Carnitine	-	-	-	-	0.035	-

Only significant *p* values are shown (<0.05).

## Data Availability

The 16S rRNA gene sequencing and metabolomic data will be deposited to Brain-CODE, https://braininstitute.ca/research-data-sharing/brain-code, accessed on 5 January 2022, a secure neuroinformatics platform for data management, sharing and analysis. While this study did not generate unreported custom codes, the codes used in the analysis are available on request from the authors.

## Supplementary Materials

The following supporting information can be downloaded at: https://www.mdpi.com/article/10.3390/ijms23063259/s1.
