# Supplementary material for: Microbe-Immune Crosstalk: Evidence That T Cells Influence the Development of the Brain Metabolome
Source: Int J Mol Sci. 2022 Mar 17;23(6):3259. doi: 10.3390/ijms23063259 (PMC8952415; doi:10.3390/ijms23063259)
Supplement: Supplementary file 1 [file ijms-23-03259-s001.zip › ijms-1567558-supplementary.pdf]

Supplementary Tables

Table S1. R^2 values for contributions of all metadata factors to beta diversity measures.

	Jaccard distance				Bray-Curtis distance				Aitchison distance			
	Cecal		Fecal		Cecal		Fecal		Cecal		Fecal	
	R^2	p	R^2	p	R^2	p	R^2	p	R^2	p	R^2	p
**Genotype**	0.265	**< 0.001**	0.254	**< 0.001**	0.228	**< 0.001**	0.251	**< 0.001**	0.263	**< 0.001**	0.263	**< 0.001**
**Age**	0.091	**< 0.001**	0.089	**< 0.001**	0.155	**< 0.001**	0.160	**< 0.001**	0.094	**< 0.001**	0.088	**< 0.001**
**Sex**	0.007	0.883	0.007	0.906	0.006	0.971	0.005	0.982	0.007	0.998	0.007	0.889

Significant results ($p < 0.01$) are bolded.

Table S2: P-values for results of Beta dispersion tests comparing Genotype groups at each timepoint in both fecal and cecal samples.

	Jaccard distance		Bray-Curtis distance		Aitchison distance	
	Cecal	Fecal	Cecal	Fecal	Cecal	Fecal
**P17**	0.301	0.461	**0.003**	0.481	0.72	0.174
**P24**	0.478	0.047	0.715	0.025	**0.001**	**0.002**
**P28**	0.106	0.244	**0.005**	0.531	0.455	**0.002**
**P84**	0.231	0.148	0.122	0.002	0.001	0.001

Significant results ($p < 0.01$) are bolded.

Table S3: Similarity matrix used to build the circos plot.

Family (ASV)		Butyrate	Valine	Isoleucine	Alanine	Acetate	Aspartate	Xylose	Maltose	Hypoxanthine	Formate	Inosine	Valerate	Acetoin	Ornithine	Malonate	Glycerophosphocholine	Glucose-6-phosphate	5-AV
Lachnospiraceae	sp19	-0.111	-0.110	-0.155	-0.098	0.092	-0.033	0.116	0.215	-0.163	-0.057	0.023	-0.227	-0.137	-0.076	-0.073	0.280	0.221	0.116
Rikenellaceae	sp8	0.523	0.336	0.455	0.132	-0.061	-0.103	-0.202	-0.287	0.106	-0.134	-0.240	**0.685**	0.432	0.312	0.229	-0.181	-0.569	-0.554
Lachnospiraceae	sp85	0.299	0.058	0.057	-0.168	0.240	-0.250	0.138	0.372	-0.427	-0.375	-0.236	0.108	0.089	0.144	0.049	**0.736**	0.023	-0.323
Lachnospiraceae	sp240	0.235	0.009	-0.011	-0.199	0.265	-0.250	0.178	0.440	-0.470	-0.377	-0.213	0.006	0.026	0.103	0.016	**0.810**	0.115	-0.255
Muribaculaceae	sp26	-0.510	-0.322	-0.434	-0.117	0.047	0.109	0.185	0.256	-0.082	0.144	0.238	**-0.655**	-0.414	-0.303	-0.220	0.138	0.539	0.540
Rikenellaceae	sp60	-0.533	-0.335	-0.453	-0.121	0.047	0.115	0.192	0.264	-0.083	0.152	0.249	**-0.683**	-0.432	-0.316	-0.229	0.140	0.561	0.565
Lachnospiraceae	sp181	-0.098	-0.097	-0.137	-0.087	0.082	-0.030	0.103	0.191	-0.145	-0.051	0.019	-0.201	-0.121	-0.067	-0.064	0.249	0.196	0.102
Lachnospiraceae	sp152	-0.223	-0.175	-0.242	-0.114	0.091	-0.002	0.146	0.250	-0.161	-0.014	0.079	-0.360	-0.222	-0.141	-0.117	0.277	0.326	0.235
Muribaculaceae	sp61	0.524	0.347	0.471	0.150	-0.081	-0.089	-0.221	-0.327	0.142	-0.113	-0.233	**0.707**	0.444	0.315	0.236	-0.242	-0.596	-0.555
Muribaculaceae	sp39	**-0.620**	-0.387	-0.523	-0.136	0.049	0.138	0.218	0.296	-0.086	0.183	0.293	**-0.789**	-0.499	-0.367	-0.265	0.145	**0.646**	**0.657**
Muribaculaceae	sp46	-0.524	-0.325	-0.438	-0.111	0.038	0.119	0.181	0.243	-0.066	0.159	0.248	**-0.662**	-0.419	-0.310	-0.223	0.111	0.540	0.555
Lachnospiraceae	sp151	0.328	0.126	0.157	-0.071	0.136	-0.186	0.033	0.159	-0.242	-0.272	-0.213	0.250	0.171	0.174	0.092	0.417	-0.136	-0.351

Lachnospiraceae	sp332	0.226	0.001	-0.022	-0.205	0.270	-0.251	0.185	0.454	-0.480	-0.379	-0.211	-0.010	0.017	0.097	0.010	**0.826**	0.130	-0.246
Ruminococcaceae	sp288	-0.006	-0.048	-0.072	-0.081	0.091	-0.062	0.086	0.180	-0.162	-0.096	-0.030	-0.102	-0.057	-0.015	-0.030	0.278	0.121	0.005
Muribaculaceae	sp105	0.556	0.354	0.478	0.134	-0.057	-0.114	-0.208	-0.291	0.100	-0.150	-0.258	**0.721**	0.455	0.331	0.242	-0.170	-0.595	-0.589
Lachnospiraceae	sp328	0.201	-0.016	-0.044	-0.212	0.274	-0.247	0.196	0.469	-0.487	-0.374	-0.200	-0.043	-0.004	0.083	-0.001	**0.838**	0.158	-0.220
Muribaculaceae	sp81	-0.488	-0.305	-0.412	-0.108	0.039	0.108	0.173	0.234	-0.069	0.144	0.230	**-0.622**	-0.393	-0.289	-0.209	0.116	0.509	0.518
Lachnospiraceae	sp122	0.444	0.277	0.373	0.096	-0.034	-0.099	-0.155	-0.210	0.060	-0.132	-0.210	0.563	0.357	0.263	0.189	-0.101	-0.461	-0.470
Muribaculaceae	sp121	0.487	0.318	0.431	0.131	-0.066	-0.089	-0.197	-0.287	0.117	-0.114	-0.220	**0.648**	0.408	0.292	0.217	-0.199	-0.542	-0.516
Muribaculaceae	sp123	0.498	0.309	0.417	0.106	-0.036	-0.113	-0.172	-0.230	0.062	-0.151	-0.236	**0.629**	0.398	0.294	0.212	-0.105	-0.513	-0.528
Muribaculaceae	sp112	**-0.607**	-0.369	-0.496	-0.115	0.027	0.150	0.194	0.249	-0.047	0.203	0.294	**-0.751**	-0.477	-0.357	-0.253	0.078	**0.606**	**0.644**
Muribaculaceae	sp96	-0.539	-0.328	-0.440	-0.101	0.024	0.133	0.172	0.220	-0.041	0.180	0.261	**-0.666**	-0.423	-0.317	-0.225	0.068	0.537	0.572
Muribaculaceae	sp168	-0.493	-0.310	-0.418	-0.111	0.042	0.107	0.177	0.242	-0.074	0.142	0.231	**-0.630**	-0.399	-0.292	-0.212	0.125	0.517	0.522
Muribaculaceae	sp237	0.488	0.314	0.425	0.124	-0.057	-0.095	-0.189	-0.270	0.101	-0.124	-0.224	**0.640**	0.404	0.291	0.214	-0.171	-0.532	-0.517
Muribaculaceae	sp172	0.514	0.298	0.398	0.071	0.007	-0.148	-0.137	-0.152	0.014	-0.204	-0.260	**0.605**	0.387	0.299	0.206	0.026	-0.475	-0.546
Muribaculaceae	sp137	-0.551	-0.341	-0.460	-0.115	0.037	0.127	0.188	0.251	-0.065	0.170	0.262	**-0.694**	-0.440	-0.326	-0.234	0.109	0.566	0.584
Ruminococcaceae	sp270	0.488	0.316	0.429	0.128	-0.062	-0.092	-0.194	-0.279	0.110	-0.119	-0.222	**0.645**	0.406	0.292	0.216	-0.186	-0.538	-0.517
Lachnospiraceae	sp418	0.217	0.026	0.017	-0.151	0.208	-0.205	0.131	0.335	-0.370	-0.308	-0.184	0.044	0.046	0.100	0.026	**0.637**	0.059	-0.236
Lachnospiraceae	sp365	-0.210	-0.161	-0.222	-0.100	0.078	0.004	0.130	0.220	-0.138	-0.004	0.077	-0.330	-0.204	-0.132	-0.108	0.236	0.296	0.221
Muribaculaceae	sp261	0.550	0.346	0.467	0.125	-0.048	-0.119	-0.198	-0.271	0.084	-0.158	-0.258	**0.704**	0.445	0.326	0.237	-0.142	-0.578	-0.583
Lachnospiraceae	sp629	0.215	-0.008	-0.034	-0.211	0.275	-0.251	0.193	0.467	-0.489	-0.380	-0.207	-0.028	0.006	0.091	0.005	**0.842**	0.147	-0.235
Muribaculaceae	sp234	0.501	0.306	0.412	0.098	-0.027	-0.120	-0.164	-0.214	0.046	-0.162	-0.241	**0.624**	0.396	0.295	0.210	-0.077	-0.505	-0.531
Peptococcaceae	sp266	0.524	0.334	0.451	0.127	-0.055	-0.107	-0.197	-0.276	0.096	-0.141	-0.242	**0.680**	0.429	0.312	0.228	-0.163	-0.562	-0.555
Peptococcaceae	sp342	-0.520	-0.339	-0.460	-0.140	0.071	0.095	0.210	0.306	-0.125	0.122	0.235	**-0.692**	-0.436	-0.312	-0.231	0.212	0.579	0.551
Peptococcaceae	sp327	-0.510	-0.338	-0.459	-0.147	0.080	0.086	0.216	0.321	-0.141	0.108	0.226	**-0.690**	-0.433	-0.307	-0.230	0.240	0.581	0.540
Ruminococcaceae	sp304	0.528	0.325	0.438	0.108	-0.033	-0.123	-0.178	-0.235	0.058	-0.165	-0.252	**0.662**	0.420	0.311	0.223	-0.097	-0.538	-0.559
Lachnospiraceae	sp889	-0.046	-0.078	-0.114	-0.100	0.105	-0.061	0.110	0.220	-0.187	-0.096	-0.015	-0.164	-0.095	-0.040	-0.050	0.321	0.177	0.047
Lachnospiraceae	sp758	0.215	-0.008	-0.034	-0.211	0.275	-0.251	0.193	0.467	-0.489	-0.380	-0.207	-0.028	0.006	0.091	0.005	**0.842**	0.147	-0.235
Ruminococcaceae	sp308	-0.540	-0.335	-0.452	-0.114	0.038	0.123	0.186	0.249	-0.067	0.165	0.256	**-0.682**	-0.432	-0.319	-0.230	0.112	0.556	0.572
Muribaculaceae	sp805	0.023	-0.025	-0.040	-0.066	0.079	-0.062	0.066	0.146	-0.140	-0.095	-0.040	-0.053	-0.027	0.004	-0.014	0.241	0.078	-0.027
Defluviitaleaceae	sp546	-0.172	-0.147	-0.204	-0.109	0.095	-0.018	0.135	0.240	-0.168	-0.037	0.052	-0.302	-0.185	-0.112	-0.098	0.288	0.282	0.180
Lachnospiraceae	sp1196	-0.049	-0.080	-0.116	-0.100	0.105	-0.060	0.110	0.221	-0.187	-0.096	-0.014	-0.167	-0.097	-0.042	-0.051	0.321	0.179	0.049
Ruminococcaceae	sp518	0.398	0.160	0.201	-0.073	0.150	-0.215	0.027	0.163	-0.266	-0.314	-0.253	0.319	0.216	0.213	0.116	0.460	-0.184	-0.426
Clostridiales_vadinBB60_group	sp935	-0.106	-0.112	-0.159	-0.107	0.103	-0.042	0.124	0.235	-0.182	-0.071	0.016	-0.233	-0.140	-0.075	-0.074	0.313	0.230	0.111
Lachnospiraceae	sp1005	-0.046	-0.078	-0.114	-0.100	0.105	-0.061	0.110	0.220	-0.187	-0.096	-0.015	-0.164	-0.095	-0.040	-0.050	0.321	0.177	0.047
Lachnospiraceae	sp813	0.230	0.033	0.026	-0.151	0.209	-0.210	0.128	0.334	-0.372	-0.315	-0.191	0.058	0.055	0.108	0.030	**0.641**	0.049	-0.249
Lachnospiraceae	sp877	0.215	-0.008	-0.034	-0.211	0.275	-0.251	0.193	0.467	-0.489	-0.380	-0.207	-0.028	0.006	0.091	0.005	**0.842**	0.147	-0.235
Lachnospiraceae	sp1102	-0.046	-0.078	-0.114	-0.100	0.105	-0.061	0.110	0.220	-0.187	-0.096	-0.015	-0.164	-0.095	-0.040	-0.050	0.321	0.177	0.047
Lachnospiraceae	sp892	0.215	-0.008	-0.034	-0.211	0.275	-0.251	0.193	0.467	-0.489	-0.380	-0.207	-0.028	0.006	0.091	0.005	**0.842**	0.147	-0.235
Lachnospiraceae	sp693	0.125	-0.038	-0.071	-0.184	0.229	-0.195	0.176	0.407	-0.408	-0.297	-0.146	-0.087	-0.036	0.044	-0.018	**0.702**	0.173	-0.138
Clostridiales_vadinBB60_group	sp834	0.215	-0.008	-0.034	-0.211	0.275	-0.251	0.193	0.467	-0.489	-0.380	-0.207	-0.028	0.006	0.091	0.005	**0.842**	0.147	-0.235
Ruminococcaceae	sp842	0.220	0.029	0.021	-0.150	0.206	-0.205	0.128	0.331	-0.367	-0.308	-0.185	0.050	0.049	0.102	0.027	**0.632**	0.054	-0.239
Erysipelotrichaceae	sp927	0.169	0.045	0.051	-0.073	0.111	-0.125	0.055	0.162	-0.198	-0.185	-0.125	0.087	0.065	0.085	0.035	0.341	-0.018	-0.182
Lachnospiraceae	sp969	0.215	-0.008	-0.034	-0.211	0.275	-0.251	0.193	0.467	-0.489	-0.380	-0.207	-0.028	0.006	0.091	0.005	**0.842**	0.147	-0.235
Lachnospiraceae	sp1393	-0.046	-0.078	-0.114	-0.100	0.105	-0.061	0.110	0.220	-0.187	-0.096	-0.015	-0.164	-0.095	-0.040	-0.050	0.321	0.177	0.047
Lachnospiraceae	sp1566	-0.046	-0.078	-0.114	-0.100	0.105	-0.061	0.110	0.220	-0.187	-0.096	-0.015	-0.164	-0.095	-0.040	-0.050	0.321	0.177	0.047
Ruminococcaceae	sp1284	-0.019	-0.058	-0.087	-0.089	0.097	-0.063	0.095	0.196	-0.173	-0.099	-0.026	-0.123	-0.070	-0.023	-0.037	0.297	0.141	0.018
Lachnospiraceae	sp1612	-0.046	-0.078	-0.114	-0.100	0.105	-0.061	0.110	0.220	-0.187	-0.096	-0.015	-0.164	-0.095	-0.040	-0.050	0.321	0.177	0.047

**Lachnospiraceae**	**sp1216**	0.215	-0.008	-0.034	-0.211	0.275	-0.251	0.193	0.467	-0.489	-0.380	-0.207	-0.028	0.006	0.091	0.005	**0.842**	0.147	-0.235
**Lachnospiraceae**	**sp1275**	0.215	-0.008	-0.034	-0.211	0.275	-0.251	0.193	0.467	-0.489	-0.380	-0.207	-0.028	0.006	0.091	0.005	**0.842**	0.147	-0.235
**Clostridiales_vadinBB60_group**	**sp980**	0.222	0.016	0.001	-0.175	0.235	-0.226	0.155	0.387	-0.418	-0.340	-0.196	0.022	0.034	0.100	0.019	**0.721**	0.090	-0.241
**Ruminococcaceae**	**sp882**	0.123	-0.022	-0.046	-0.152	0.193	-0.169	0.143	0.337	-0.343	-0.257	-0.132	-0.052	-0.017	0.048	-0.008	0.591	0.129	-0.136
**Eubacteriaceae**	**sp1095**	-0.145	-0.127	-0.178	-0.099	0.087	-0.021	0.121	0.217	-0.155	-0.039	0.041	-0.262	-0.160	-0.095	-0.085	0.265	0.247	0.152
**Clostridiales_vadinBB60_group**	**sp947**	0.252	0.107	0.136	-0.036	0.083	-0.128	0.006	0.081	-0.148	-0.187	-0.156	0.214	0.144	0.136	0.077	0.257	-0.132	-0.270
**Lachnospiraceae**	**sp1852**	-0.046	-0.078	-0.114	-0.100	0.105	-0.061	0.110	0.220	-0.187	-0.096	-0.015	-0.164	-0.095	-0.040	-0.050	0.321	0.177	0.047
**Lachnospiraceae**	**sp1853**	-0.046	-0.078	-0.114	-0.100	0.105	-0.061	0.110	0.220	-0.187	-0.096	-0.015	-0.164	-0.095	-0.040	-0.050	0.321	0.177	0.047
**Ruminococcaceae**	**sp1037**	0.201	0.062	0.073	-0.071	0.115	-0.136	0.049	0.159	-0.204	-0.201	-0.142	0.121	0.087	0.103	0.047	0.352	-0.044	-0.217
**Ruminococcaceae**	**sp1111**	0.241	0.049	0.049	-0.131	0.189	-0.198	0.107	0.291	-0.336	-0.297	-0.189	0.092	0.074	0.116	0.041	0.579	0.013	-0.260
**Lachnospiraceae**	**sp1752**	0.011	-0.031	-0.048	-0.067	0.077	-0.057	0.068	0.147	-0.138	-0.088	-0.034	-0.067	-0.036	-0.003	-0.019	0.237	0.088	-0.014
**Lachnospiraceae**	**sp1308**	0.215	-0.008	-0.034	-0.211	0.275	-0.251	0.193	0.467	-0.489	-0.380	-0.207	-0.028	0.006	0.091	0.005	**0.842**	0.147	-0.235
**Rikenellaceae**	**sp1058**	0.197	0.029	0.023	-0.128	0.178	-0.179	0.109	0.284	-0.317	-0.269	-0.163	0.051	0.048	0.092	0.026	0.546	0.040	-0.214
**Ruminococcaceae**	**sp1303**	0.239	0.016	-0.001	-0.190	0.255	-0.244	0.168	0.420	-0.453	-0.367	-0.211	0.022	0.035	0.107	0.020	**0.780**	0.098	-0.259
**Muribaculaceae**	**sp1711**	0.215	-0.008	-0.034	-0.211	0.275	-0.251	0.193	0.467	-0.489	-0.380	-0.207	-0.028	0.006	0.091	0.005	**0.842**	0.147	-0.235
**Muribaculaceae**	**sp1905**	0.215	-0.008	-0.034	-0.211	0.275	-0.251	0.193	0.467	-0.489	-0.380	-0.207	-0.028	0.006	0.091	0.005	**0.842**	0.147	-0.235
**Lachnospiraceae**	**sp1906**	0.226	0.027	0.018	-0.157	0.216	-0.213	0.135	0.348	-0.384	-0.321	-0.191	0.047	0.048	0.105	0.027	**0.661**	0.061	-0.245
**Ruminococcaceae**	**sp1907**	0.208	0.015	0.001	-0.163	0.219	-0.210	0.144	0.361	-0.390	-0.317	-0.183	0.021	0.032	0.093	0.018	**0.671**	0.083	-0.226
**Lachnospiraceae**	**sp2064**	0.215	-0.008	-0.034	-0.211	0.275	-0.251	0.193	0.467	-0.489	-0.380	-0.207	-0.028	0.006	0.091	0.005	**0.842**	0.147	-0.235
**Lachnospiraceae**	**sp2065**	0.215	-0.008	-0.034	-0.211	0.275	-0.251	0.193	0.467	-0.489	-0.380	-0.207	-0.028	0.006	0.091	0.005	**0.842**	0.147	-0.235
**Lachnospiraceae**	**sp2066**	0.215	-0.008	-0.034	-0.211	0.275	-0.251	0.193	0.467	-0.489	-0.380	-0.207	-0.028	0.006	0.091	0.005	**0.842**	0.147	-0.235
**Lachnospiraceae**	**sp2067**	0.215	-0.008	-0.034	-0.211	0.275	-0.251	0.193	0.467	-0.489	-0.380	-0.207	-0.028	0.006	0.091	0.005	**0.842**	0.147	-0.235
**Lachnospiraceae**	**sp2068**	0.215	-0.008	-0.034	-0.211	0.275	-0.251	0.193	0.467	-0.489	-0.380	-0.207	-0.028	0.006	0.091	0.005	**0.842**	0.147	-0.235
**Lachnospiraceae**	**sp2069**	0.215	-0.008	-0.034	-0.211	0.275	-0.251	0.193	0.467	-0.489	-0.380	-0.207	-0.028	0.006	0.091	0.005	**0.842**	0.147	-0.235
**Lachnospiraceae**	**sp2070**	0.215	-0.008	-0.034	-0.211	0.275	-0.251	0.193	0.467	-0.489	-0.380	-0.207	-0.028	0.006	0.091	0.005	**0.842**	0.147	-0.235

The similarity matrix is calculated as the correlation between the variables' loadings, and is an approximation of Pearson correlation. Positive (> 0.6) and negative (< -0.6) correlations are indicated by red and black squares, respectively.

Supplementary Figures

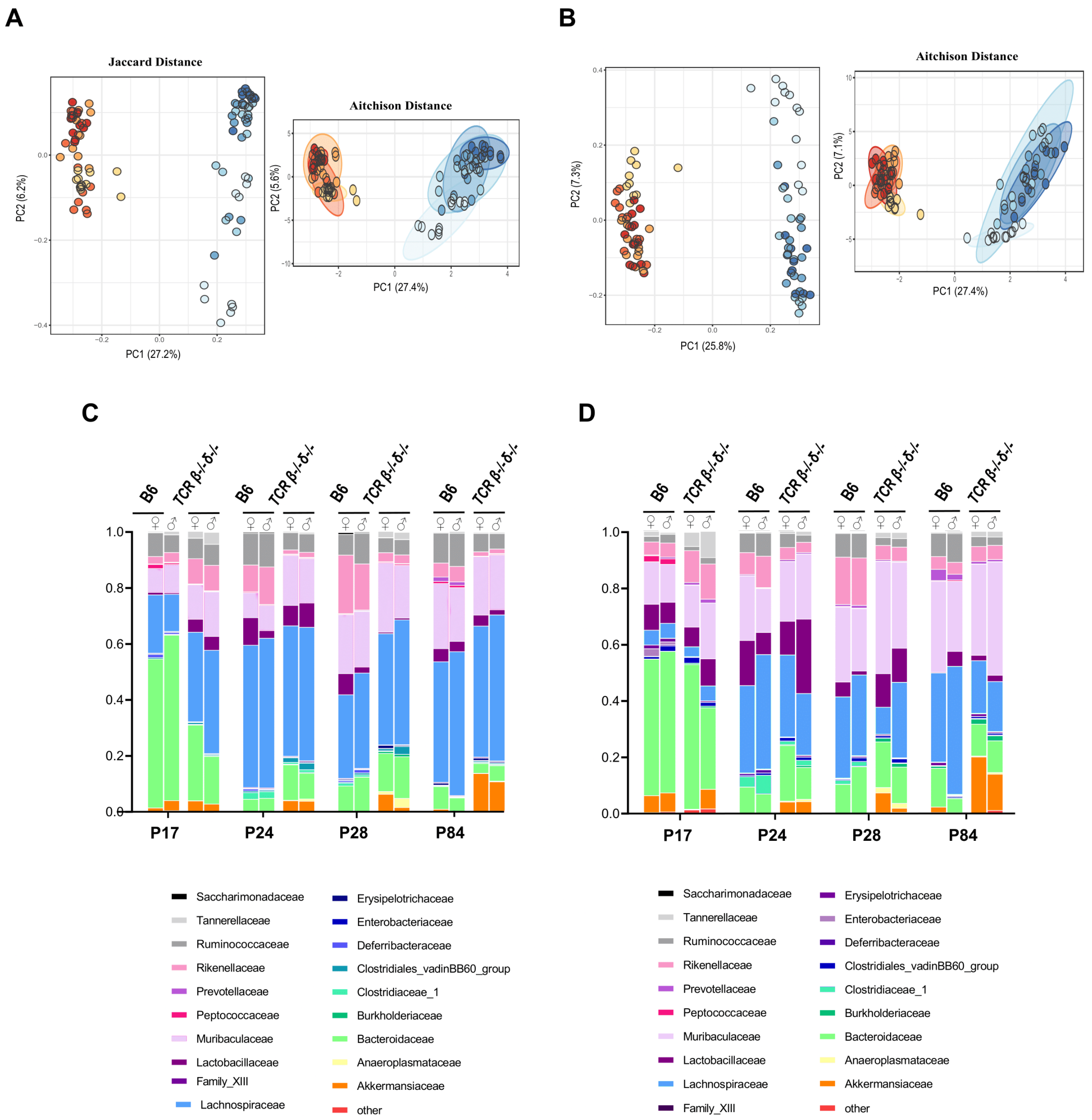

Supplemental Figure S1. *TCR β-/-δ-/-* mice exhibit compositional changes in the microbiome. Upper row shows the first two principal components of Jaccard and Aitchison distance matrices for (**A**) cecal and (**B**) fecal samples. Bottom row shows the family-level distribution barplots for cecal (**C**) and (**D**) fecal samples, averaged across each group at each postnatal timepoint. Only the top 20 families are displayed.

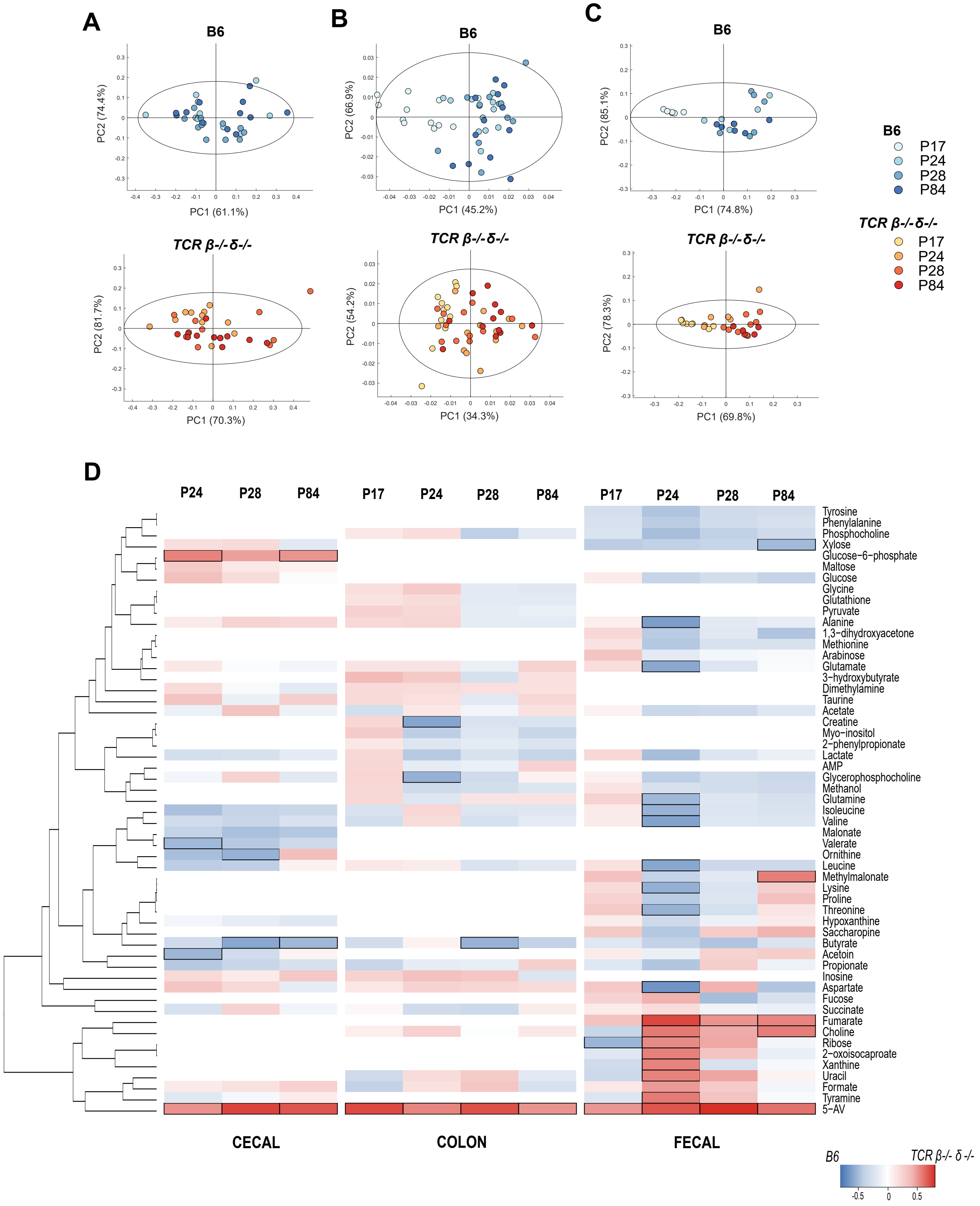

Supplemental Figure S2. Metabolic variation of cecal, colon and fecal samples associated with age. PCA scores of (**A**) cecal, (**B**) colon and (**C**) fecal samples from B6 (left) and *TCR β-/-δ-/-* (right) mice colored by age. In cecal samples from both B6 and TCR β-/-δ-/- mice, the effect of age was very weak. In colon and fecal samples obtained from B6 mice, a clear separation can be observed between B6 mice at P17 and B6 mice at later ages (P24-84), while this metabolic difference is no longer evident in mice depleted of T-cells. (**D**) OPLS-DA models constructed separately on the metabolic profiles of the B6 and *TCR β-/-δ-/-* mice using age as the outcome variable allowed the differences in the age-related metabolic changes to be studied in the presence and absence of T-cells. The heatmap illustrates the

correlation coefficient (r) obtained from OPLS models constructed against age for each genotype. In red are metabolites that are positively associated with age, in blue those negatively associated with age. Black squares indicate correlations with $r > |0.5|$. Increasing age was associated with an elevation in cecal ornithine and colonic myo-inositol in B6, but not *TCR β-/-δ-/-* mice. However, in *TCR β-/-δ-/-* mice xylose was observed to decreased in the cecum with age while cecal choline increased. Such changes were not observed in B6 mice. *TCR β-/-δ-/-* mice also exhibited a decrease in fecal tyramine, phenylalanine, hypoxanthine, formate and an increase in lactate with age, which was not observed in B6 mice.

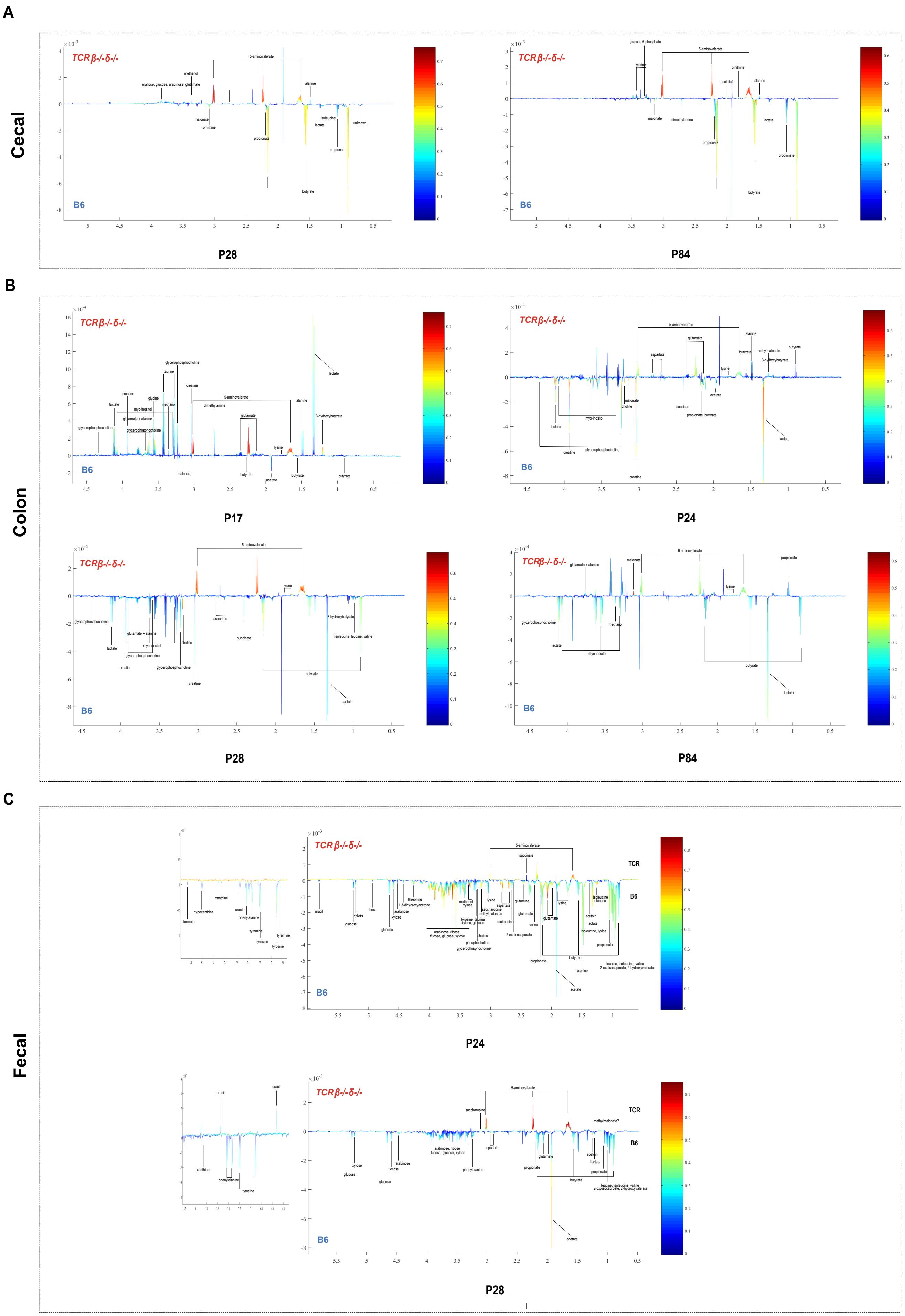
A
Cecal
TCRβ-/-δ-/-
B6
P28
P84
B
Colon
P17
P24
P28
P84
C
Fecal
TCR
B6
P24
P28

Supplemental Figure S3. OPLS-DA correlation plots showing the metabolic variation between B6 and *TCR β-/-δ-/-* mice. OPLS models were constructed on the (A) cecal, (B) colon and (C) fecal ^{1}H nuclear magnetic resonance spectroscopic profiles of age-matched B6 and *TCR β-/-δ-/-* mice, with genotype as the response variable. The plots show the contribution of each metabolite to the sample classification (*i.e.* loadings). Peaks pointing up represent those metabolites that are more abundant in *TCR β-/-δ-/-* mice, while peaks pointing down are metabolites that are more abundant in B6 mice. The Yrepresents arbitrary units (a.u.). The color of the peaks indicates the magnitude of the correlation between that metabolite and class membership, with red indicating a strong correlation and blue indicating a weak correlation. Only significant models are shown.

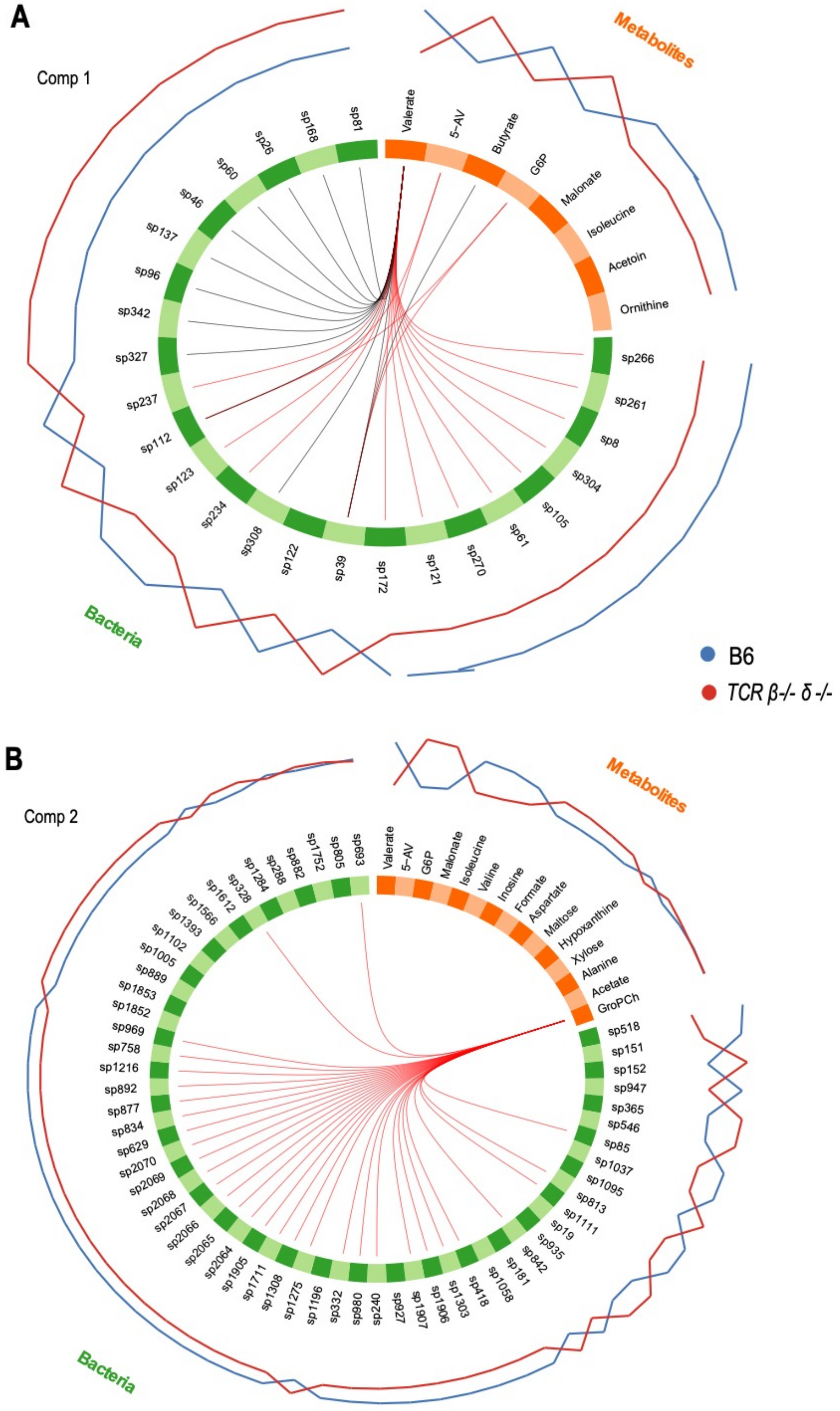

Supplemental Figure S4. Circos plots for each individual component. These plots allow for the visualization of the relationships between variables (lines within the circle), as well as the expression levels of each variable in *TCR β-/-δ-/-* and B6 groups (lines outside of the circle, red for *TCR β-/-δ-/-* and blue for B6) for principal component 1 (**A**) and 2 (**B**). Each variable is colored based on which biological block it belongs to (*metabolites* in blue, *bacteria* in green). The graph only shows correlations greater than 0.6 (in red) or lower than -0.6 (in black).

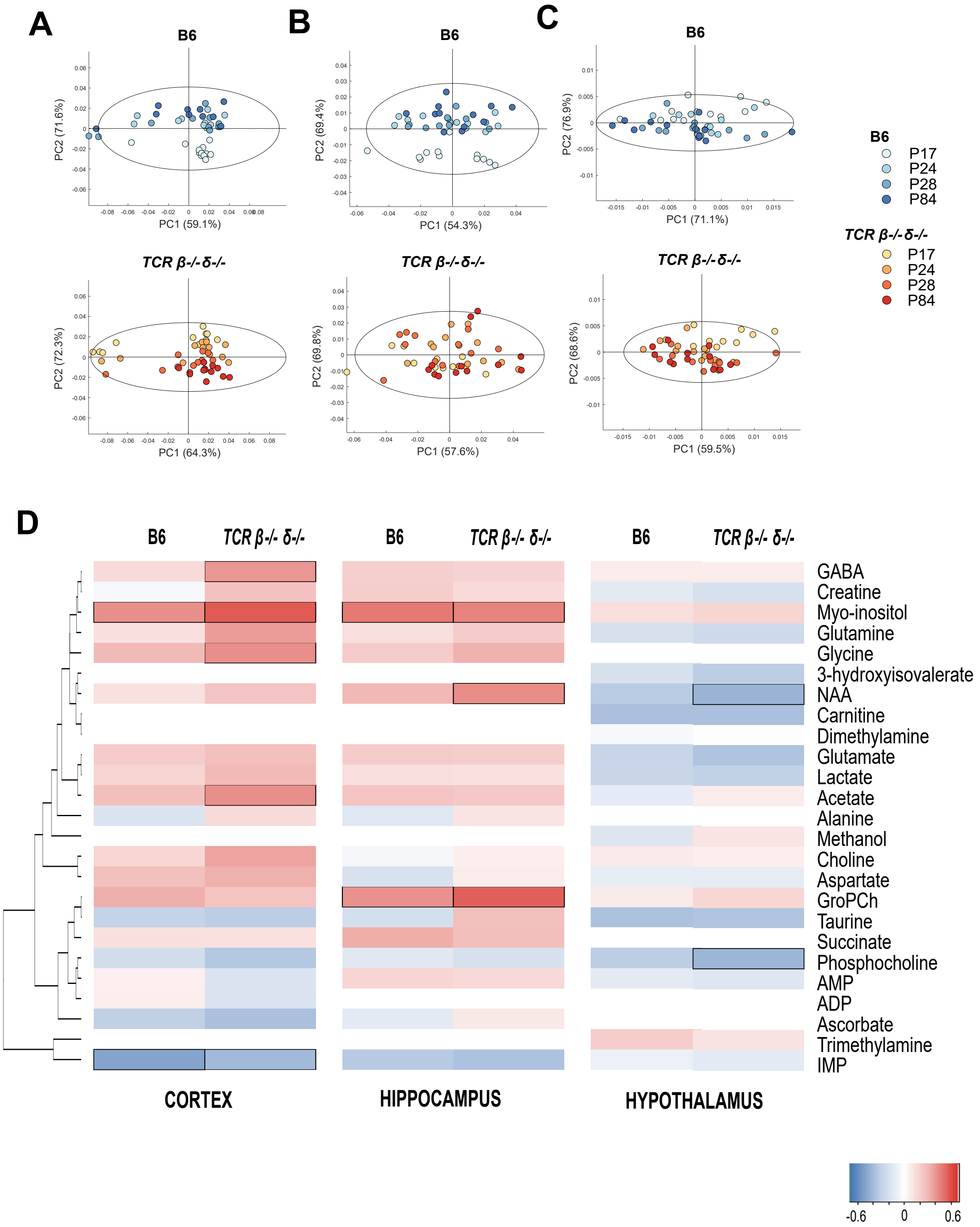

Supplemental Figure S5. Metabolic variation of cortical, hippocampal and hypothalamic samples associated with age. PCA scores of (A) cortical, (B) hippocampal and (C) hypothalamic samples from B6 (left) and *TCR β-/-δ-/-* (right) mice colored by age. (D) The age-related metabolic changes for each genotype and sample type were assessed by constructing OPLS-DA models (for B6 and *TCR β-/-δ-/-* mice separately). The heatmap illustrates the correlation coefficient (r) obtained from OPLS models constructed against age for each genotype. In red are metabolites that are positively associated with age, in blue those negatively associated with age. Black squares indicate correlations with r > │0.4│. An age-related increase in GABA, glycine and acetate was observed in the cortex of *TCR β-/-δ-/-*mice, but not in B6 mice. *N*-Acetyl-aspartate (NAA) increased with age in the hippocampus, while it decreased with age in the hypothalamus of *TCR β-/-δ-/-*mice only. Similarly, only *TCR β-/-δ-/-*mice showed an age-associated decrease in hypothalamic phosphocholine.

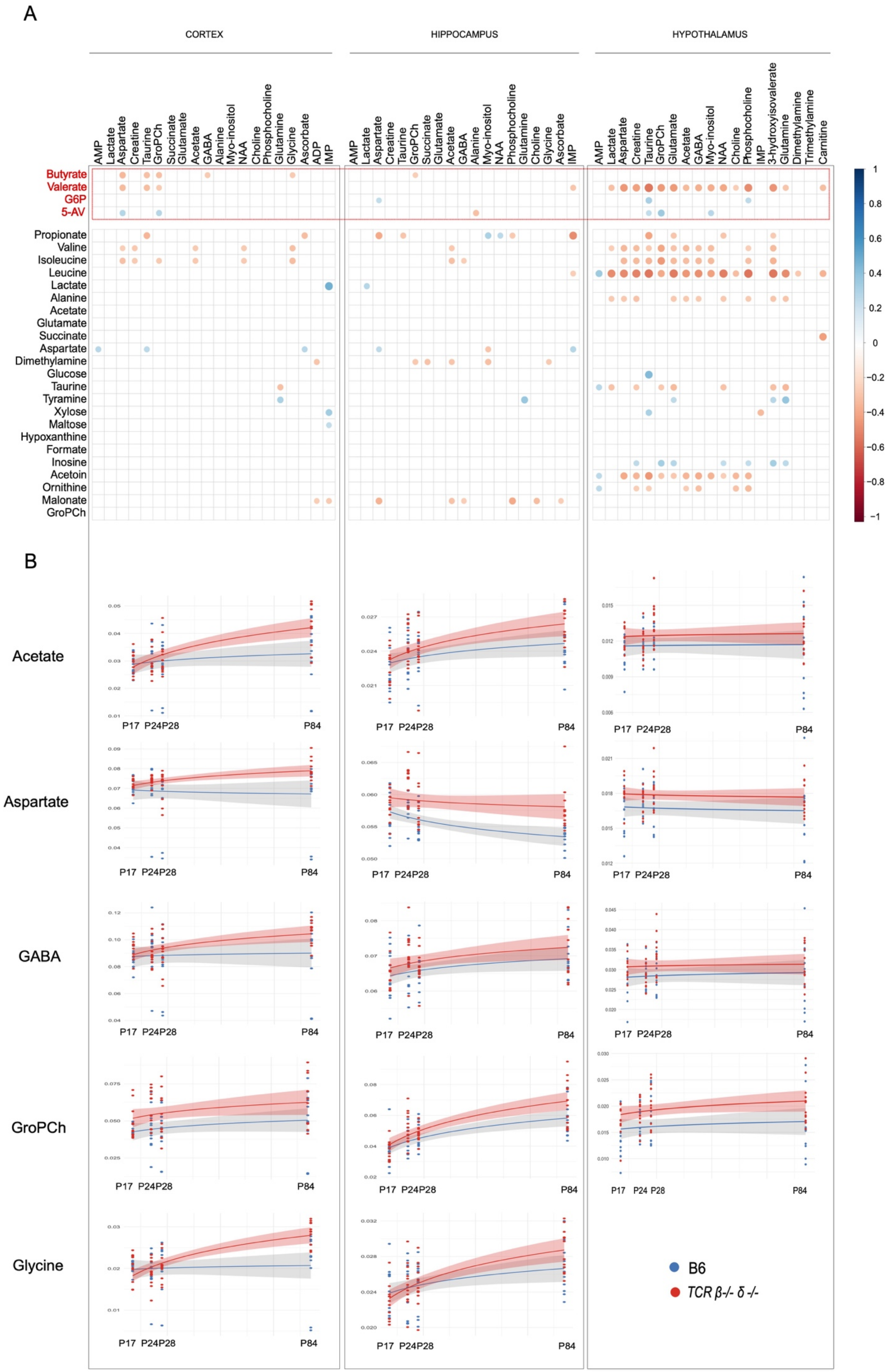
A
CORTEX
HIPPOCAMPUS
HYPOTHALAMUS
AMP
Lactate
Aspartate
Creatine
Taurine
GroPCh
Succinate
Glutamate
Acetate
GABA
Alanine
Myo-inositol
NAA
Choline
Phosphocholine
Glutamine
Glycine
Ascorbate
ADP
IMP
3-hydroxyisovalerate
Dimethylamine
Trimethylamine
Carnitine
Butyrate
Valerate
G6P
5-AV
Propionate
Valine
Isoleucine
Leucine
Lactate
Alanine
Acetate
Glutamate
Succinate
Aspartate
Dimethylamine
Glucose
Taurine
Tyramine
Xylose
Maltose
Hypoxanthine
Formate
Inosine
Acetoin
Ornithine
Malonate
GroPCh
1
0.8
0.6
0.4
0.2
0
−0.2
−0.4
−0.6
−0.8
−1
B
Acetate
Aspartate
GABA
GroPCh
Glycine
P17
P24P28
P84
B6
TCR β-/- δ -/-

Supplemental Figure S6. Genotype differences in developmental trajectories of neuroactive metabolites in brain tissue. (A) Correlogram depicting the Pearson correlation coefficients between the abundance of cecal and brain (cortical, hippocampal and hypothalamic) metabolites. In the cortex, aspartate, taurine, glycerophosphocholine, GABA and glycine were negatively correlated with the expression of butyrate in the cecum. Cortical aspartate, taurine and glycerophosphocholine were negatively associated with cecal valerate, while cortical aspartate and glycerophosphocholine were positively associated with cecal 5-aminovalerate. In the hippocampus, a positive correlation emerged between aspartate and cecal glucose-6-phosphate, and a negative association between alanine and cecal 5-aminovalerate and between IMP and cecal valerate. The hypothalamus showed the highest number of significant associations, with 15/20 metabolites exhibiting a negative correlation with cecal valerate. These included aspartate, taurine, glycerophosphocholine, glutamate, acetate, GABA, myo-inositol and phosphocholine. In the hypothalamus, taurine and phosphocholine were also positively associated with cecal glucose-6-phosphate, while taurine, myo-inositol and glycerophosphocholine were positively associated with cecal 5-aminovalerate. (B) Line plots showing the developmental trajectories of cortical, hippocampal and hypothalamic metabolites across the four developmental timepoints (P17, P24, P28, P84) for B6 (blue) and *TCR β-/-δ-/-*mice (red). Across brain samples, the inhibitory neurotransmitter GABA and amino acid glycine, as well as the short-chain fatty acid acetate and the membrane component glycerophosphocholine exhibited a steep increase with age in *TCR β-/-δ-/-*mice, while they were relatively stable in B6 mice. At P84, these metabolites were elevated in *TCR β-/-δ-/* compared to B6 mice. While the excitatory amino acid aspartate was also elevated in *TCR β-/-δ-/-*mice relative to B6, its trajectory was different in the cortex compared to hippocampus and hypothalamus. In the cortex, aspartate increased slightly with age in *TCR β-/-δ-/-*mice, while it remained stable in B6 mice. In the hippocampus and hypothalamus, its concentrations decreased in both genotypes, but was elevated in *TCR β-/-δ-/-*mice compared to B6 at all timepoints. Plots were created by fitting a linear model with formula y ~ log(x), where x = age and y = metabolite abundance. Shaded areas represent the 95% confidence interval.
